# VDAC1 at the crossroads of cell metabolism, apoptosis and cell stress

**DOI:** 10.15698/cst2017.10.104

**Published:** 2017-10-01

**Authors:** Varda Shoshan-Barmatz, Eduardo N. Maldonado, Yakov Krelin

**Affiliations:** 1 Department of Life Sciences and the National Institute for Biotechnology in the Negev, Ben-Gurion University of the Negev, Beer-Sheva, 84105, Israel.; 2 Department of Drug Discovery & Biomedical Sciences, Medical University of South Carolina, Charleston, SC, USA.

**Keywords:** apoptosis, cancer, metabolism, mitochondria, VDAC1

## Abstract

This review presents current knowledge related to VDAC1 as a multi-functional mitochondrial protein acting on both sides of the coin, regulating cell life and death, and highlighting these functions in relation to disease. It is now recognized that VDAC1 plays a crucial role in regulating the metabolic and energetic functions of mitochondria. The location of VDAC1 at the outer mitochondrial membrane (OMM) allows the control of metabolic cross-talk between mitochondria and the rest of the cell and also enables interaction of VDAC1 with proteins involved in metabolic and survival pathways. Along with regulating cellular energy production and metabolism, VDAC1 is also involved in the process of mitochondria-mediated apoptosis by mediating the release of apoptotic proteins and interacting with anti-apoptotic proteins. VDAC1 functions in the release of apoptotic proteins located in the mitochondrial intermembrane space via oligomerization to form a large channel that allows passage of cytochrome c and AIF and their release to the cytosol, subsequently resulting in apoptotic cell death. VDAC1 also regulates apoptosis via interactions with apoptosis regulatory proteins, such as hexokinase, Bcl2 and Bcl-xL, some of which are also highly expressed in many cancers. This review also provides insight into VDAC1 function in Ca^2+^ homeostasis, oxidative stress, and presents VDAC1 as a hub protein interacting with over 100 proteins. Such interactions enable VDAC1 to mediate and regulate the integration of mitochondrial functions with cellular activities. VDAC1 can thus be considered as standing at the crossroads between mitochondrial metabolite transport and apoptosis and hence represents an emerging cancer drug target.

## VDAC ISOFORMS, STRUCTURE, AND CHANNEL ACTIVITY

### VDAC isoforms and cellular localization

Three different VDAC isoforms, VDAC1, VDAC2 and VDAC3, sharing ~70% identity and structural and some functional properties 1,2, are expressed in mammalian mitochondria, with VDAC1 being the major protein expressed. However, significantly differences in the functions of the three isoforms were found 1,3,4, suggesting they assume different physiological roles 1,5. The three isoforms are expressed in most tissue types, with VDAC1 expression being higher than that of VDAC2 and VDAC3 in most but not all tissues 1,2.

Both VDAC1- and VDAC3-deficient mice are viable. However, *VDAC1^−/−^* mice (inbred C57BL/6 background) were born in less than expected numbers according to the Mendelian ratio, suggesting partial embryonic lethality. Studies using *VDAC1^−/−^* mice confirmed the importance of this protein as a carrier of metabolites across the OMM 6. In mice, deletion of VDAC1 and VDAC2 reduces respiratory capacity 7, and the absence of VDAC3 causes male sterility, while a lack of both VDAC1 and VDAC3 causes growth retardation 8 and is associated with deficits in learning behavior and synaptic plasticity 9. In this review, the focus will be on the VDAC1 isoform.

Using various approaches, VDAC was detected not only in the mitochondria but also in other cell compartments 3, such as the plasma membrane 3,10, including the caveolae and caveolae-like domains 11, the sarcoplasmic reticulum (SR) of skeletal muscles 12, and the ER of rat cerebellum 13,14. A possible mechanism for targeting VDAC protein to the plasma membrane proposes that this version of the protein contains an N-terminal signal peptide responsible for targeting to the cell membrane 15,16. The exact function of extra-mitochondrial VDAC is unknown, although several possible roles have been proposed (reviewed in 17).

### VDAC1 structure, channel conductance, properties and regulation

**Figure 1:**
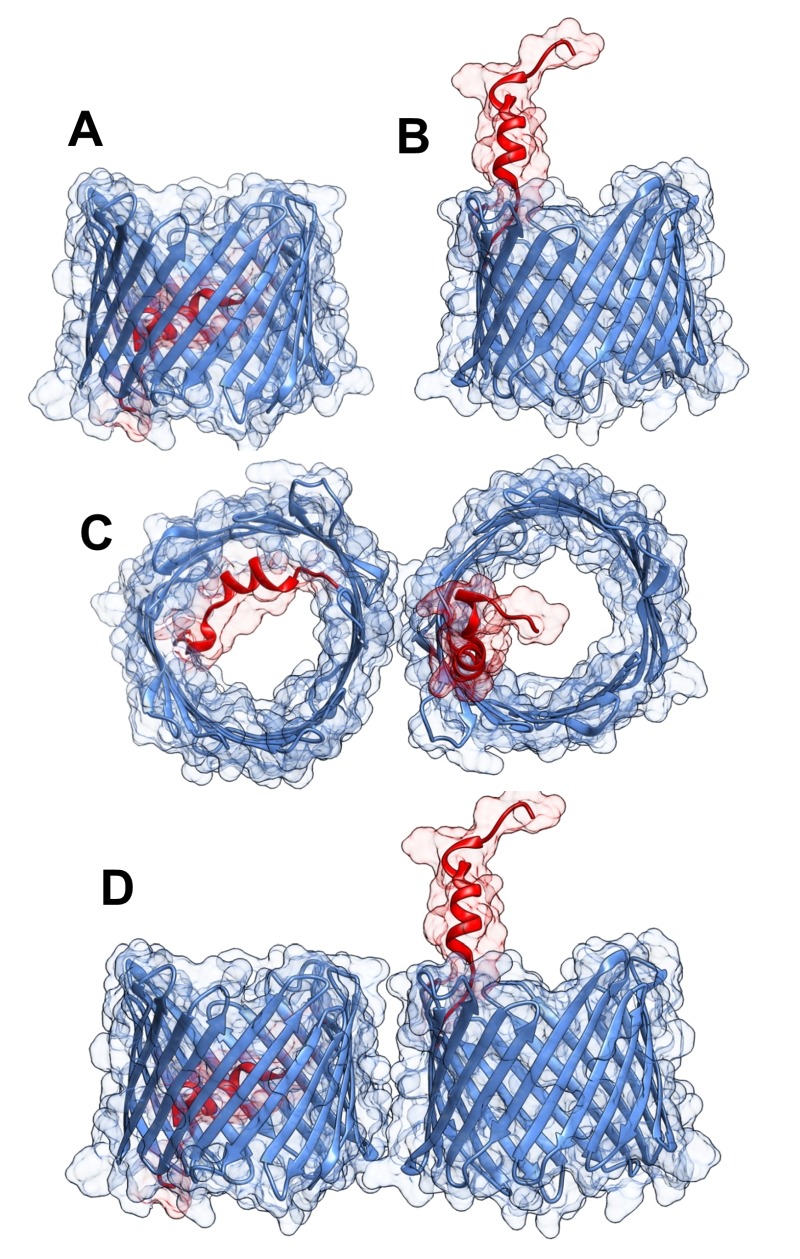
FIGURE 1: Three-dimensional structure of VDAC1. VDAC1 monomer and dimer structures. **(A)** Side-view of the crystal structure of VDAC1 (PDB code: 3EMN). The β-barrel is formed by 19 β strands and the N-terminal domain (colored red) is folded into the pore interior. **(B)** A proposed model for the conformation of VDAC1 with its N-terminal on the outside of the VDAC1 pore. **(C) **Top-view of VDAC1 dimer with the N-terminal helix nested inside the VDAC1 pore in one monomer and outside of the pore in the other. **(D) **Side-view of proposed dimer of VDAC1. Figures were prepared using PyMOL software.

The three-dimensional structure of VDAC isoform 1 was determined at atomic resolution, revealing that VDAC1 is composed of 19 transmembrane β-strands connected by flexible loops to form a β-barrel, with strands β1 and β19 being in parallel conformation along with a 25-residue-long N-terminal region that lies inside the pore 18,19,20 (**Fig. 1A**). The N-terminal region is proposed to move in the open space 21 and translocate from the internal pore to the channel surface 22 (**Fig. 1B**). This segment is ideally positioned to regulate the conductance of ions and metabolites passing through the VDAC1 pore 20,18.

The pore diameter of the channel has been estimated to be between 3 and 3.8 nm 18, and is decreased to about 1.5 nm when the N-terminal α-helix is located within the pore 18,19,20. The stretch of multiple glycine residues (^21^GlyTyrGlyPheGly^25^) 1,5 connecting the N-terminal domain to β-strand 1 of the barrel is thought to provide the flexibility required for N-terminal region translocation out of the internal pore of the channel 22. The reported results suggest that the N-terminal region mobility is involved in channel gating, interaction with anti-apoptotic proteins, and VDAC1 dimer formation 22, as well as serving the interaction site of apoptosis-regulating proteins of the Bcl-2 family (i.e., Bax, Bcl-2, and Bcl-xL) 22,23,24,25,26 and hexokinase (HK) 23,27.

Purified and membrane-embedded VDAC1 is able to assemble into dimers, trimers, tetramers, hexamers, and higher-order moieties 1,28,29,30,31,32,33,34,35,36. The contact sites between VDAC1 molecules in dimers and higher oligomers were identified 37. Under physiological conditions, VDAC1 is present as a monomer and dimer, with a contact site involving β-strands 1, 2, and 19. However, upon apoptosis induction, VDAC1 dimers undergo conformational changes to assemble into higher oligomeric states with contact sites also involving β-strands 8 and 16 37. VDAC1 oligomerization has been proposed to play important physiological roles in the regulation of VDAC1 function, including contributing to stabilizing the protein 38, serving as a platform for other proteins that oligomerize, such as HK 36 and creatine kinase 39, and finally, in mediating Cytochrome *c* release and the binding of apoptosis-regulating proteins 23,28,36 (see below).

VDAC1 has been purified from mitochondria isolated from liver, brain, and other tissues 40, and its channel properties were characterized following reconstitution into a planar lipid bilayer (PLB). Such bilayer-reconstituted VDAC1 assumes a variety of voltage-dependent conducting states, with different selectivities and permeabilities. VDAC1 shows symmetrical bell-shaped voltage-dependent conductance. At low voltages (-20 to +20 mV), VDAC1 exists in a high conductive state (~4 nS at 1 M KCl), and shows a preference for transporting anions over cations, while at high positive or negative potentials (> 40 mV), VDAC1 switches to lower conductance states permeable to small ions 41,42. VDAC1 is permeable to small ions (e.g. Cl-, K^+^, Na^+^), and also to large anions, such as glutamate 41 and ATP 43, and to large cations, such as acetylcholine and dopamine 41.

The interactions of VDAC1 with Ca^2+^, ATP, glutamate, NADH, and different proteins were suggested to modulate its activity 44,45,46,47. VDAC1 has been shown to be phosphorylated by protein kinase A (PKA) 48 and protein kinase C (PKC)ε 49, and VDAC1 and VDAC2 were found to be phosphorylated at a particular Tyr residue under hypoxic conditions 50.

## VDAC1, A MULTI-FUNCTIONAL CHANNEL CONTROLLING CELL ENERGY AND METABOLISM

**Figure 2:**
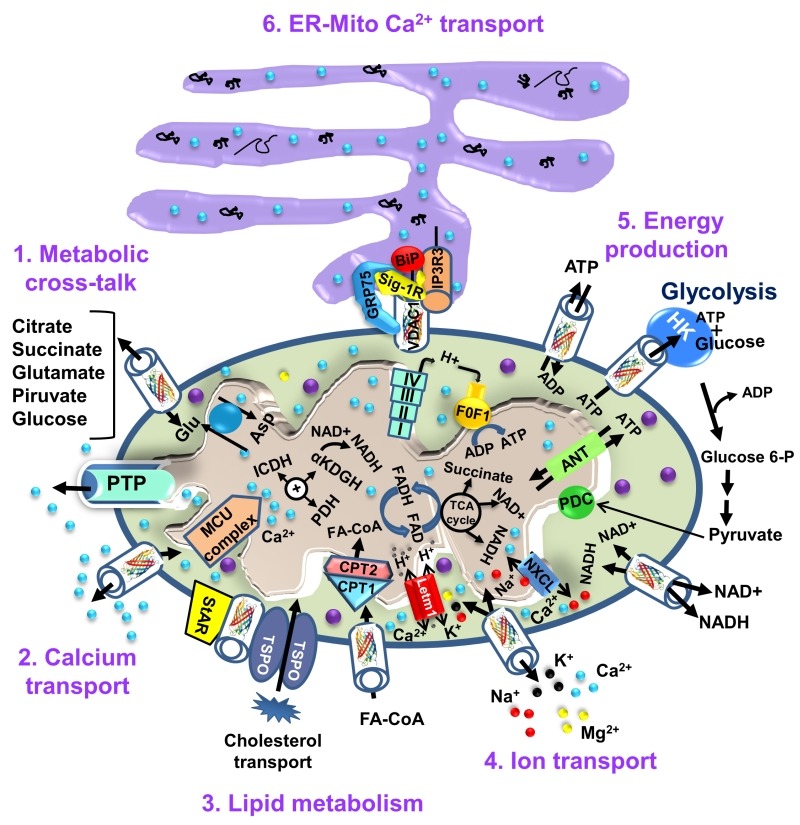
FIGURE 2: VDAC1 as a multi-functional channel involved in metabolite, cholesterol and Ca^2+^ transport, energy production and in ER-mitochondria structural and functional association. The functions of VDAC1 in cell life include control of the metabolic cross-talk between the mitochondria and the rest of the cell energy production, regulation of glycolysis via binding of HK, Ca^2+^ signaling and cholesterol transport. The various functions of VDAC1 in the cell and mitochondria functions are presented. These include: **1**. Control of the metabolic cross-talk between mitochondria and the rest of the cell; **2**. Transport of Ca^2+ ^to and from the IMS and acting in Ca^2+^ signaling; **3.** Lipid metabolism; **4**. Transport of ions, such as Mg^2+^, Zn^+^, Na^+^ and K^+^; **5**. Mediating cellular energy production by transporting ATP/ADP and NAD^+^/NADH and acyl-CoA (FA-CoA) from the cytosol to and from the IMS, and regulating glycolysis via association with HK; **6**. Structurally and functionally contributing to ER-mitochondria contacts, mediating Ca^2+ ^transport from the ER to mitochondria. Ca^2+^ influx and efflux systems in the IMM are shown. The mitochondrial Ca^2‏+^ uniporter (MCU), in association with a calcium-sensing accessory subunit (MCU1), mediates Ca^2+^ transport from the IMS into the matrix. The ryanodine receptor (RyR) in the IMM mediates Ca^2+^ influx. NCLX, a Na^+^/Ca^2+ ^exchanger, mediates Ca^2+^ efflux from the matrix to the IMS. High levels of matrix Ca^2‏+^ trigger the opening of the PTP, a fast Ca^2+^ release channel. Molecular fluxes are indicated by arrows. The function of Ca^2+^ in regulating energy production is mediated via activation of the TCA cycle enzymes pyruvate dehydrogenase (PDH), isocitrate dehydrogenase (ICDH) and α-ketoglutarate dehydrogenase (α-KGDH), leading to enhanced activity of the TCA cycle. The electron transport chain (ETC) and the ATP synthase (F_o_F_1_) are also presented. VDAC1 mediates the transfer of acyl-CoAs across the OMM to the IMS, where they are converted into acylcarnitine by CPT1a for further processing by β-oxidation. VDAC1 is involved in cholesterol transport by being a constituent of a multi-protein complex, the transduceosome, containing StAR/TSPO/VDAC1.The ER-mitochondria association is presented with key proteins indicated. These include the inositol 3 phosphate receptor type 3 (IP_3_R3), the sigma 1 receptor (Sig1R) (a reticular chaperone), binding immunoglobulin protein (BiP), the ER HSP70 chaperone, and glucose-regulated protein 75 (GRP75). IP_3_ activates IP_3_R in the ER to release Ca^2+^ that is directly transferred to the mitochondria via VDAC1.

The OMM, the interface between the cytosol and a mitochondrion, is also a limiting boundary for modulating cell bioenergetics, mediated via VDAC1. The metabolites and ions that reach the matrix must first cross the OMM to reach the mitochondrial intermembrane space (IMS), from where they are then transported by about 53 secondary transport proteins called mitochondrial carriers (MCs). The mitochondrial carrier proteins of the family SLC25 (solute carrier family 25, located in the inner mitochondrial membrane (IMM)) are substrate-specific and mediate electrochemical, chemical and membrane potential gradient-dependent transport. The SLC25 family includes carriers for Pi (PiC), ADP/ATP (ANT) and aspartate/glutamate, pyruvate, acyl carnitine, oxoglutarate, and citrate, among others 51. On the other hand, VDAC1 is the sole channel mediating the flux of ions, nucleotides and other metabolites up to ~5,000 Da (e.g. pyruvate, malate, succinate, NADH/NAD^+^) across the OMM, as well as hemes and cholesterol 1,52. Thus, at the OMM, VDAC1 is perfectly positioned to function as gatekeeper for the entry and exit of substrates and products into and out of the mitochondria, and to interact with proteins that mediate and regulate the integration of mitochondrial functions with other cellular activities 1,29,30,30,31,42,53,54 (**Fig. 2**).

VDAC1 allows the shuttling of ATP/ADP and NAD^+^/NADH, with mitochondria-generated ATP being transported to the cytosol in exchange for ADP, which is utilized in oxidative phosphorylation (OXPHOS) to generate ATP. As such, VDAC1 controls the electron transport chain 1 (**Fig. 2**), as well as the normal flow of metabolites 55. The importance of VDAC1 in channeling ATP from the mitochondria to kinases has been presented in several studies. These showed that VDAC1 interacts with HK and creatine kinase (CrK) to convert newly generated ATP into high-energy storage forms, like glucose-6-phosphate (G-6-P) and creatine phosphate in brain and muscle, respectively. The interaction of VDAC1 with HK mediates coupling between OXPHOS and glycolysis, while at the contact sites between the IMM and OMM, VDAC1 forms a complex with the adenine nucleotide translocase (ANT), and CrK 56. Dimeric αβ-tubulin was proposed as a regulator of VDAC1 permeability to ATP, with heterodimers of αβ-tubulin decreasing the passage of ATP through the channel 57. The importance of VDAC1 in cell energy and metabolism homeostasis is reflected in the findings that closure of VDAC 55 or down-regulation of VDAC1 expression decreased metabolite exchange between mitochondria and the cytosol and inhibited cell growth 58,59. Moreover, VDAC1 is overexpressed in many cancer cells 32, as discussed below.

Cholesterol is another metabolite transported across the OMM 60 (**Fig. 2**), with VDAC1 being considered as a necessary component of a multi-protein complex, the transduceosome, involved in the process. In addition to VDAC1, the transduceosome also includes the OMM high-affinity cholesterol-binding protein translocator protein (TSPO) and the steroidogenic acute regulatory protein (StAR) 61 (**Fig. 2**). Cholesterol synthesis is highly elevated in various cancer cells, with hepatocellular carcinoma cells containing 2-10-fold more mitochondrial cholesterol (mainly in the OMM) than found in liver mitochondria 62. The increased binding of HK to the mitochondria may increase synthesis and uptake of cholesterol into the mitochondria of cancer cells 63. At high levels, cholesterol can reduce the activity of membrane-associated proteins and thus inhibit the metabolic functions of VDAC1 64. As such, VDAC1 is involved in cholesterol synthesis and transport, as well as being subject to cholesterol-mediated regulation.

Finally, it appears that VDAC1 is also part of a complex mediating the transport of fatty acids through the OMM in rat liver mitochondria 65. In this case, it is hypothesized that VDAC1 acts as an anchor, linking the long-chain acyl-CoA synthetases (ACSLs) at the OMM to carnitine palmitoyltransferase 1a (CPT1a), which faces the IMS. According to the proposed model, upon activation by ACSL, VDAC1 transfers acyl-CoAs across the OMM to the IMS, where they are converted into acylcarnitine by CPT1a. Moreover, recently it was shown that fatty acid accumulation in hepatocytes leads to a lack of phosphorylation by GSK-3β, indicating interplay between lipids and VDAC function 66. Furthermore, it was recently proposed that VDAC behaves as a lipid sensor 67.

## CANCER, METABOLISM, MITOCHONDRIA, AND VDAC1 

One of the main functions of the telomere is to prevent the metabolic reprogramming in cancer cells that require plasticity of the metabolic machinery, regardless of cellular or tissue origin, a critical process that promotes cell proliferation with alterations seen in the metabolism of several substrates, including glucose and glutamine 68,69. In the 1920’s , Otto Warburg demonstrated increased lactic acid production resulting from high glycolysis in tumors, as compared to non-proliferating cells. The Warburg effect described a metabolic phenotype characterized by enhanced glycolysis and suppression of mitochondrial metabolism at any level of oxygen. However, although enhanced glycolysis is a prominent feature of most tumor cells, the mitochondria of cancer cells maintain a membrane potential, oxidize respiratory substrates, and generate NADH and ATP, among other functional parameters 70,71,72,73. The view of cancer as a metabolic disease that originated with the experiments of Otto Warburg was gradually displaced by the concept of cancer as a genetic disease. Recently, evidence supporting a general hypothesis that genomic instability and essentially all hallmarks of cancer, including aerobic glycolysis, can be linked to impaired mitochondrial function and energy metabolism, has been reviewed 74,75. Interestingly, no specific gene mutations or chromosomal abnormalities are common to all cancers 76, while nearly all cancers display aerobic glycolysis, regardless of their tissue or cellular origin. The view of cancer as primarily being a metabolic disease will impact approaches to cancer management and prevention.

Malignant cancer cells typically display high rates of glycolysis, even when fully oxygenated (aerobic glycolysis), and an altered redox balance 77,78,79. To increase glycolysis, cancer cells up-regulate the transcription of genes involved in the glycolytic pathway (i.e., glucose transporters, glycolytic enzymes, etc.). Cancer cells in fact use both glycolysis and OXPHOS, with the ratio depending on the prevalent normoxic or hypoxic environmental conditions and their capacity to express adequate levels of oncogenes and tumor suppressor gene products for cell growth 80. By regulating the metabolic and energetic functions of mitochondria, VDAC1 can, therefore, control the fate of cancer cells. Mitochondrial-bound HK, considered the rate-limiting enzyme of glycolysis, is over-expressed in cancer 1,81,82. The association of HK with VDAC1 offers several advantages to cancer cells 1,32, such as direct access to mitochondrial sources of ATP, assumption of the role of an anti-apoptotic protein, reducing intracellular levels of reactive oxygen species (ROS) and increasing synthesis and uptake of cholesterol. The HK-VDAC1 complex formation is regulated by Akt 83 and glycogen synthase kinase 3 beta (GSK3β), while the HK-VDAC complex is disrupted by VDAC phosphorylation 84.

**Figure 3:**
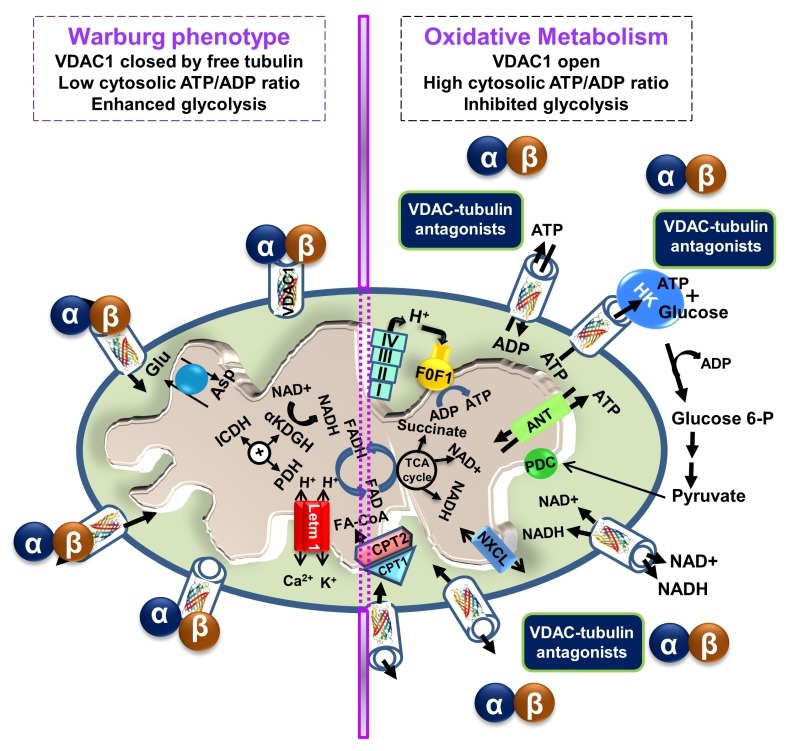
FIGURE 3: VDAC1-tubulin interaction: a metabolic switch to modulate mitochondrial metabolism in cancer cells. In cancer cells, high levels of free tubulin close VDAC1, decreasing the flux of metabolites, ATP and ADP through the OMM. VDAC1 closing leads to low generation of mitochondrial ATP and subsequently, to a low cytosolic ATP/ADP ratio that favors glycolysis in the Warburg phenotype. Erastin, a VDAC-tubulin antagonist, opens VDAC1 by blocking the inhibitory effect of free tubulin. VDAC1 opening leads to increased mitochondrial metabolism and to a high cytosolic ATP/ADP ratio that inhibits glycolysis and reverts the Warburg phenotype. αβ indicates tubulin heterodimers.

In recent years, cumulative evidence indicates that free tubulin in cancers cells interacts with VDAC 70,85. Dimeric αβ-tubulin decreases the conductance of bilayer-reconstituted VDAC1 and VDAC2 and also decreases respiration in cardiac myocytes and isolated brain mitochondria 86,87. In cancer cells, microtubule destabilization induced by colchicine or microtubule stabilization by paclitaxel increased and decreased free tubulin, leading to decreased and increased ΔΨm, respectively 70. The dynamic regulation of ΔΨm by free tubulin appears to occur only in cancer cells. It has been proposed that the dynamic changes of ΔΨm brought about by free tubulin in tumor cells are related to αβ-tubulin heterodimers modulating VDAC conductance (**Fig. 3**) 70.

VDAC1 and VDAC2 isolated after VDAC2/3 or VDAC1/3 double knockdown in cancer cells were shown to be sensitive to tubulin inhibition. Even more, VDAC1 knockdown in tumor cells decreased ΔΨm, indicating that VDAC1 is critical for the maintenance of ΔΨm and is regulated by endogenous free tubulin 85. Inhibition of VDAC1 conductance by free tubulin is a main contributor to the suppression of mitochondrial metabolism in the Warburg phenotype. Recently, the VDAC-tubulin interaction was proposed to serve as a metabolic switch to increase or decrease mitochondrial metabolism, ATP generation and cytosolic ATP/ADP ratios 88. High and low cytosolic ATP/ADP ratios inhibit or favor aerobic glycolysis, respectively. Thus, blockage of the inhibitory effect of tubulin on VDAC by VDAC-tubulin antagonists promotes mitochondrial metabolism and reverses the Warburg phenotype (**Fig. 3**). The VDAC-tubulin interaction represents a new pharmacological target for the development of novel anti-cancer agents 88.

### Silencing VDAC1 expression reduces cell energy homeostasis, inhibiting cells and tumor growth 

As cellular metabolic and energy reprogramming are cancer hallmarks essential for tumor progression, and VDAC1 is a key regulator of these processes 1,30,32,47,52,89, down-regulation of VDAC1 expression is expected to impact cancer cell growth. VDAC1 down-regulation results in reduced metabolite exchange between the mitochondria and the cytosol, leading to inhibited cell growth. Indeed, silencing VDAC1 expression reduced cellular ATP levels and cell growth, with tight correlation between cell growth and cellular ATP levels being seen 58. shRNA directed against hVDAC1 inhibited the development of a HeLa cervical tumor 90. Nano-molar concentrations of a single siRNA specific to human VDAC1 silenced VDAC1 expression and inhibited the growth of various cancer cell types. In fact, such treatment inhibited solid tumor development and growth in lung cancers (over 90%) both *in vitro* and *in vivo* 59.

**Figure 4:**
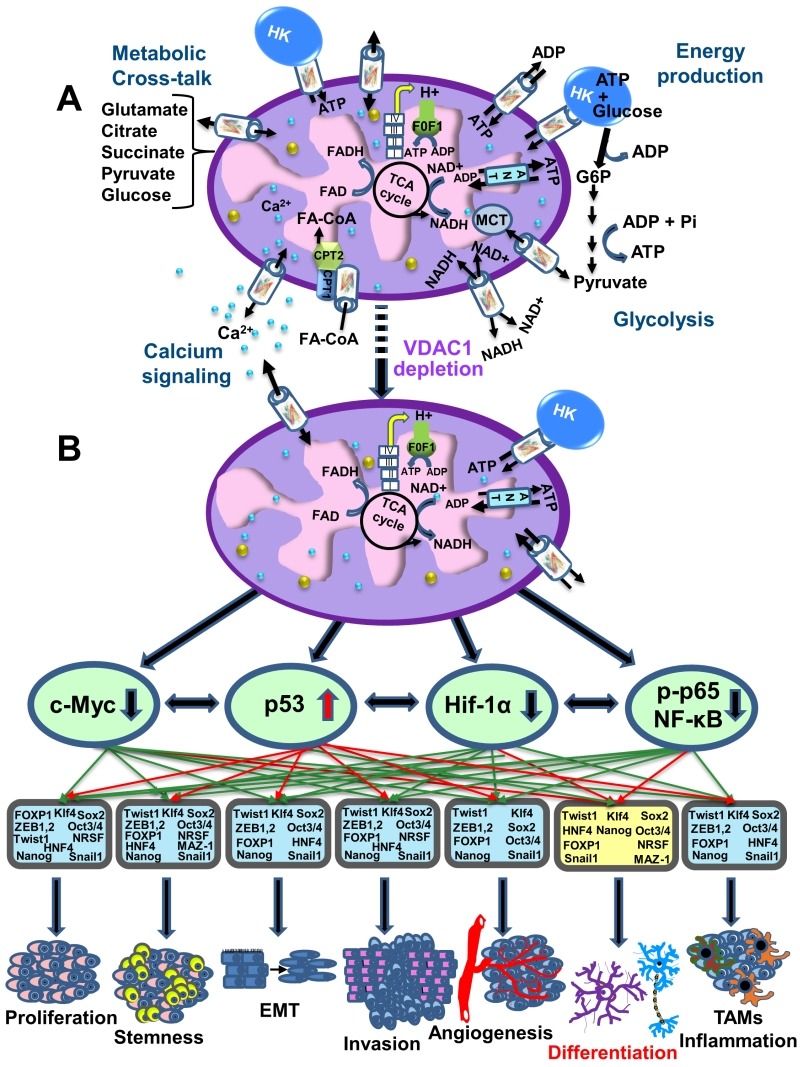
FIGURE 4: VDAC1-depletion and metabolic reprogramming leading to alterations in key transcription factor levels and biological processes: a reversal of oncogenic properties and cell differentiation. **(A) **A schematic presentation of mitochondria in a cancer cell before treatment with hVDAC1 siRNA. Here, cancer cells maintain homeostatic energy and metabolic states, with HK bound to VDAC1 accelerating glycolysis and mitochondrial function to allow sufficient ATP and metabolite precursor levels to support cell growth and survival. (**B)** VDAC1 depletion leads to dramatic decreases in energy and metabolite generation. This leads to changes in master metabolism regulator (p53, HIF1-α, c-Myc and NF-kb, P65) expression levels, which alters the expression of transcription factors associated with stemness, EMT, cell proliferation, invasion, TAMs and angiogenesis, while leading to differentiation into astrocyte- or neuron-like cells.

Recently, a global change in tumor hallmarks upon silencing VDAC1 expression was demonstrated in glioblastoma multiform (GBM) 91. Using a sub-cutaneous or an intracranial-orthotopic GBM model, we demonstrated that si-VDAC1 inhibited tumor growth, with the residual tumor showing reversed oncogenic properties, such as reprogramed metabolism, angiogenesis, epithelial-mesenchymal transition (EMT), invasiveness and stemness, leading to differentiation into neuron- and astrocyte-like cells 91 (**Fig. 4**). These VDAC1 depletion-mediated effects involved alterations in transcription factors (TFs) that regulate signaling pathways associated with cancer hallmarks, allowing for attacks on the interplay between metabolism and oncogenic signaling networks (to be explored here), leading to cancer stem cell (CSC) differentiation into neuronal-like cells 91.

### microRNA-mediated regulation of VDAC1

A number of microRNAs (miRNAs) targeting VDAC1 were reported and found to be modified under pathological conditions. miR-29a 92 and miR-320a 93 have been shown to reduce VDAC1 expression levels. Another miRNA species, miR-7, was shown to inhibit VDAC1 expression, proliferation and metastasis in hepatocellular carcinoma 94, possibly by affecting the permeability transition pore (PTP) 95. Recently, lncRNA-H19/miR-675 was reported to regulate high glucose-induced apoptosis by targeting VDAC1, and thus provides a novel therapeutic strategy for the treatment of diabetic cardiomyopathy 96.

The therapeutic potential of a number of miRNAs able to regulate VDAC1 expression levels is clear in view of the observation that VDAC1 over-expression is associated with a variety of pathological conditions, including Alzheimer's disease (AD) 97,98,99, and cardiovascular diseases (CVDs) 100. In addition, hyperglycemia has been shown to increase VDAC1 expression in β-cells 101 and in the kidney 102.

## VDAC1 AS A PLAYER IN MITOCHONDRIA-MEDIATED APOPTOSIS

**Figure 5:**
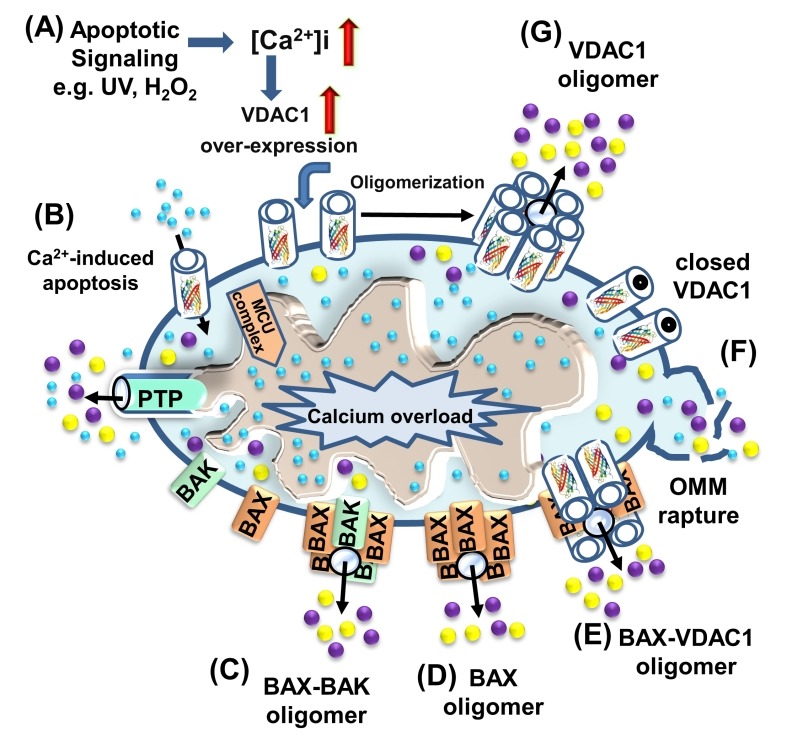
FIGURE 5: VDAC1 function in cell death, with apoptosis inducers enhancing VDAC1 expression levels and oligomerization. A schematic representation of VDAC1 function in cell death - Different models for the release of apoptogenic proteins, such as Cyto *c* (purple) and AIF (yellow), are shown. **(A)** Proposed model suggesting that apoptotic stimuli or conditions cause enhanced VDAC1 expression via increases in [Ca^2+^]i levels or transcription factors, leading to activation of the VDAC1 promoter. The increase in VDAC1 expression shifts the equilibrium towards the VDAC1 oligomeric state, forming a hydrophilic protein-conducting channel capable of mediating the release of apoptogenic proteins (e.g., Cyto *c* and AIF) from the mitochondrial IMS to the cytosol. **(B)** Mitochondrial Ca^2+^ overload induces apoptosis. Ca^2+^ transport across the OMM, as mediated by VDAC1, and then across the IMM, as mediated by the MCU, leads to Ca^2+^ overload in the matrix. This, in turn, causes dissipation of the membrane potential, mitochondrial swelling, PTP opening, Cyto *c*/AIF release and the triggering of apoptotic cell death. **(C)** Bax/Bak oligomerization and activation, forming a route for Cyto *c*/AIF release. **(D)** Bax activation leads to its association with the OMM, followed by its oligomerization as a large oligomer/complex, forming a Cyto *c*/AIF-conducting channel. **(E)** The interaction of the pro-apoptotic protein Bax with VDAC1 forms hetro-oligomers that mediate Cyto c/AIF release. **(F)** Prolonged VDAC1 closure leads to mitochondrial matrix swelling and OMM rupture, resulting in the appearance of a non-specific release pathway for apoptogenic proteins.

Programmed cell death, or apoptosis, is the biological process by which a cell rapidly proceeds towards death upon receiving specific stimuli. The apoptotic machinery in humans consists of a molecular network comprising a large number of proteins that regulate a cascade of events leading to apoptosis through multiple parallel pathways. It is well accepted that mitochondria serve as integrators and amplifiers of apoptosis by mediating and regulating the release of pro-apoptotic proteins and/or disrupting cellular energy metabolism 103. Upon transfer of an apoptotic signal into the cell, mitochondrial membrane permeability changes so as to facilitate the release of apoptogenic proteins, such as cytochrome *c* (Cyto *c*), apoptosis-inducing factor (AIF), and SMAC/Diablo, from the IMS into the cytosol 54,103. These proteins participate in complex processes, resulting in the activation of proteases and nucleases, leading to protein and DNA degradation and cell death. However, it remains unclear how these apoptotic initiators cross the OMM and are thus released into the cytosol. Several hypotheses regarding the mechanism of mitochondria-mediated apoptosis have been proposed (Fig. 5) (for reviews, see 1,30,104). The major models include OMM rupture and non-specific release of IMS proteins into the cytosol 55,105,106, opening of the PTP in response to over-production of ROS or Ca^2+^ overload 107, a large channel formed by Bax and/or Bak oligomers 108,109, a channel formed by hetero-oligomers of VDAC1 and Bax 110,111 or VDAC1 oligomers (**Fig. 5**) 23,28,29,30,31,36,104,112,113.

All of the apoptotic proteins known to translocate to the cytoplasm following an apoptotic stimulus reside in IMS. Thus, only the permeability of the OMM needs to be modified for their release 114,115,116,117. Hence, VDAC1, as an OMM channel, could mediate Cyto *c* release. Indeed, VDAC1 is now accepted as a key player in mitochondria-mediated apoptosis, with VDAC1 silencing or over-expression affecting apoptosis induction 1,23,33,118,119,120,121,122. Exogenous over-expression of VDAC from various sources was found to induce apoptotic cell death regardless of cell type 113,118,119,120,121,122. VDAC1 over-expression-induced cell death was prevented by RuR 122,123, Bcl2, DIDS 120 or by over-expression of HK-I 118,122,124, with all these agents directly interacting with VDAC1. Finally, reducing VDAC1 expression by siRNA efficiently prevented cisplatin-induced apoptosis and Bax activation in non-small cell lung cancer (NSCLC) cells 125, and inhibited selenite-induced PTP opening in HeLa cells 126. VDAC1-siRNA also attenuated endostatin-induced apoptosis 127. In addition to the evidence above, release of Cyto *c* via purified VDAC reconstituted into Cyto *c*-encapsulating liposomes has been demonstrated 36,128,129. It is thus proposed that VDAC1 oligomerization is a key step in the release of the pro-apoptotic proteins from the IMS to the cytosol 23,28-31, 36, 104, 112, 113.

### A VDAC1 oligomeric structure as a Cyto *c* release pathway 

When considering models of VDAC1-mediated protein release, the molecular sizes of the released proteins (12 to 100 kDa) and the diameter of the VDAC1 pore (2.6-3.0 nm) should be considered. The VDAC1 pore can allow passage of nucleotides and small molecules but is too small for the passage of a folded protein like Cyto *c*. As such, we proposed the formation of a large protein-conducting channel within a VDAC1 homo-oligomer serving as the Cyto *c* release route. Indeed, upon apoptosis induction by various stimuli, VDAC1 undergoes conformational changes and oligomerization, followed by Cyto *c* release, and finally, apoptosis 29,33,36,118,129.

Apoptosis induction leads to VDAC1 oligomerization regardless of the cell type or apoptosis inducer used, including staurosporine (STS), curcumin, As_2_O_3_, etoposide, cisplatin, selenite, H_2_O_2_ or UV light, all affecting mitochondria yet acting via different mechanisms 28,112. Moreover, shifting the equilibrium towards the VDAC1 oligomeric state upon over-expression of the protein in the absence of apoptosis stimuli resulted in release of pro-apoptotic proteins, leading to cell death, regardless of cell type, in a manner that could be inhibited by anti-apoptotic proteins 23,33,33,113,118,119,120,121,122. The specific lipid composition of the OMM significantly enhances VDAC1 oligomerization 130, while p53 also promotes VDAC1 oligomerization 131.

Several VDAC1-interacting molecules inhibit both apoptosis and VDAC1 oligomerization as induced by various stimuli 28,104,112,113,119,120,122,132,133. These include 4,4 diisothiocyanostilbene-2,2-disulfonic acid (DIDS), 4-acetamido-4-isothiocyanato-stilbene-2,2-disulfonic acid (SITS), 4,4' diisothiocyanatodihy-drostilbene-2,2'-disulfonic acid (H2DIDS), 4,4’-dinitrostilbene-2,2’-disulfonic acid (DNDS), and diphenylamine-2-carboxylate (DPC). Similarly, the newly developed VDAC1-interacting molecules AKOS-022 and VBIT-4 prevented VDAC1 oligomerization and apoptosis as induced by various means and in several cell lines 134. These compounds also protected against apoptosis-associated mitochondrial dysfunction, specifically restoring dissipated mitochondrial membrane potential, and thus cell energy and metabolism, decreasing ROS production, and preventing disruption of intracellular Ca^2+^ levels. The use of these apoptosis inhibitors thus supports the tight coupling between VDAC1 oligomerization and apoptosis induction. Inhibiting apoptosis at an early stage via prevention of VDAC1 oligomerization may be an effective approach for blocking or slowing apoptosis in neurodegenerative disorders 135,136 and various cardiovascular diseases, where enhanced apoptosis also occurs 137,138,139.

To conclude, it is proposed that VDAC1 exists in a dynamic equilibrium between the monomeric and oligomeric states, with apoptosis inducers or VDAC1 over-expression shifting the equilibrium towards oligomerization. Thus, the cellular VDAC1 expression level and its oligomeric state are crucial factors in the process of mitochondria-mediated apoptosis.

### The mode of action of apoptotic stimuli and VDAC1 over-expression - a new concept 

Several studies have demonstrated that the induction of apoptosis by various reagents is accompanied by an increase in the level of VDAC1 expression 54. These include arbutin (hydroquinone-O-beta-D-glucopyranoside), a tyrosinase inhibitor that induces apoptosis in A375 human ma-lignant melanoma cell by causing VDAC1 over-expression 140. Up-regulation of VDAC1 expression was noted in acute lymphoblastic leukemia (ALL) cell lines following prednisolone treatment 141. Somatostatin up-regulated the expression of VDAC1 and VDAC2 in the LNCaP prostate cancer cell line 142. Up-regulation of VDAC1 expression levels was also induced by the hepatitis E virus ORF3 protein 143. Also, both UV irradiation and ROS were shown to up-regulate VDAC1 expression 144,145,146 that was prevented by the ROS chelator epigallocatechin 144. Cisplatin induced VDAC1 over-expression in a cisplatin-sensitive cervix squamous cell carcinoma cell line (A431), while down-regulation of VDAC1 was noted in a cisplatin-resistant cell line (A431/Pt) 147. Up-regulation of VDAC expression was proposed to mediate the actions of vorinostat, a histone deacetylase inhibitor that induced synergistic anti-proliferative and pro-apoptotic effects in NSCLC cells in combination with EGFR-tyrosine kinase inhibitors 148. Finally, apoptosis induction by H_2_O_2_, etoposide, cisplatin, selenite and UV irradiation all led to enhanced VDAC1 expression levels, which was accompanied by VDAC1 oligomerization, Cyto *c* release and apoptosis 54,112,113.

As apoptosis induction by agents such as STS, As_2_O_3_, and selenite disrupt [Ca^2+^]i homeostasis and energy production 112,113,149, apoptosis-induced VDAC1 up-regulation is proposed to be mediated by an increase in [Ca^2+^]i. Indeed, we have shown that pro-apoptotic-agents induce cell death through Ca2+-dependent up-regulation of VDAC1 expression levels 54,112,113. Direct elevation of [Ca2+]i by the Ca^2+^-mobilizing agents A23187, ionomycin and thapsigargin also resulted in VDAC1 over-expression, VDAC1 oligomerization and apoptosis 112,113. In contrast, decreasing [Ca^2+^]i using the cell-permeable Ca^2+^-chelating reagent BAPTA-AM inhibited VDAC1 over-expression, VDAC1 oligomerization and apoptosis. Thus, the increase in [Ca^2+^]i induced by apoptosis stimuli was found to be a pre-requisite for induction of VDAC1 over-expression and apoptosis 112,113. The over-expressed VDAC1 forms oligomers and this triggers Cyto *c* release and then cell death. The following new concept of apoptosis induction is thus proposed 112,113: Apoptosis inducers ( increased [Ca^2+^]i ( enhanced VDAC1 expression levels ( VDAC1 oligomerization ( Cyto *c* release ( apoptosis.

As such, up-regulation of the expression of VDAC1 may represent a new common mode of action of apoptosis induction.

## VDAC1 AND CA^2+^ HOMEOSTASIS

Mitochondria serve as a major hub for cellular Ca^2+^ homeostasis, regulating oxidative phosphorylation and modulating cytosolic Ca^2+^ signals of cell death and secretion 150,151. Mitochondria can rapidly sequester large amounts of Ca^2+^ at the expense of the membrane potential across the IMM and mediate Ca^2+^ efflux. Ca^2+^ is an essential co-factor for several rate-limiting TCA enzymes (i.e., pyruvate dehydrogenase, isocitrate dehydrogenase, and α-ketoglutarate dehydrogenase) located in the matrix, such that intra-mitochondrial Ca^2+^ controls energy and metabolism. To reach the matrix, Ca^2+^ must cross both the OMM and the IMM, in a manner mediated by several proteins. VDAC1 acts in the OMM, whereas the mitochondrial Ca^2+^ uniporter (MCU) 152,153 and the Na^+^/Ca^2+^ exchanger, NCLX, the major Ca^2+^ efflux mediator 154, are both found in the IMM.

The function of VDAC1 in regulation of cell Ca^2+^ homeostasis was recently summarized 155. VDAC1 in the OMM is highly Ca^2+^-permeable and transports Ca^2+^ into and out of the IMS 156,157,158,159, consequently allowing Ca^2+^ access to IMM transporters. Ruthenium red (RuR) 157,160,161, ruthenium amine binuclear complex (Ru360) 161, the photo-reactive analogue azido ruthenium (AzRu) 162 and the lanthanides La^3+^and Tb^3+^ 160 all reduce VDAC1 conductance in the case of native but not mutated VDAC1 160,161.

Competition between Ca^2+^ and RuR 160 suggests that VDAC1 possesses divalent cation-binding site(s). The physiological function of the VDAC1 Ca^2+^-binding site(s), reflected in the regulation of VDAC1 gating by physiological levels of Ca^2+^, prolongs a fully open state of the channel, thereby promoting metabolite exchange 156. Thus, it has become apparent that VDAC1 both mediates Ca^2+^ transport and is also regulated by Ca^2+^ binding.

VDAC1 also functions in the ER/mitochondria-Ca^2+^ cross-talk. VDAC1 is a constituent of a supra-molecular complex composed of the IP3 receptor in the ER and VDAC1 in the OMM, linked by a chaperone called GRP75 13,163, together with mitofusin-2 164,165.

Thus, by transporting Ca^2+^, VDAC1 plays a fundamental role in regulating mitochondrial Ca^2+^ homeostasis, oxidative phosphorylation, and Ca^2+^ crosstalk among mitochondria, cytoplasm, and the ER.

## VDAC AND OXIDATIVE STRESS 

Oxidative stress results when production of ROS exceeds the capacity of mitochondrial and cellular anti-oxidant defenses to remove these toxic species. ROS act as second messengers in cell signaling and are essential for multiple biological processes in normal cells. However, ROS are also well known contributors to cell proliferation and cell death 166,167,168,169, provoking damage to multiple cellular organelles and processes 170.

Mitochondria are the major source of ROS formation, mostly at complex I (site IQ), complex II (site IIF) and complex III (site IIIQo) 171,172,173. O_2_•- generated at complex III is released to the cytosol through VDAC1. By contrast, O_2_•- produced at complexes I and II is released to the matrix, where it is rapidly converted to H_2_O_2_ by superoxide dismutases located in the mitochondrial matrix (MnSOD or SOD2) and the cytosol (Cu,ZnSOD or SOD1) 174. H_2_O_2_ acts as a cell signaling molecule that does not disrupt redox homeostasis 175 and modulates the pro-survival PI3K/Akt/mTOR HIF-1 and MAP/ERK pathways to promote tumorigenesis and metastasis 176,177,178. H_2_O_2_ also forms the highly reactive hydroxyl radical (OH•) by the Fenton reaction. Whereas both H_2_O_2_ and O_2_•- react with mitochondrial and extra-mitochondrial structures, OH• is so reactive that its effects are almost completely restricted to mitochondria. O_2_•- and OH• can inactivate mitochondrial proteins, and damage mitochondrial DNA and lipids in the MIM 179,180. Cytosolic ROS also activate members of the MAPK family of serine/threonine kinases, especially the c-Jun N-terminal kinase (JNK), the extracellular signal-regulated kinase (ERK 1/2), and p38, whose signaling can cause mitochondrial dysfunction. JNK translocation to mitochondria has been shown to cause mitochondrial dysfunction in several models 181,182.

Cells possess several anti-oxidant defense mechanisms, including the presence of various endogenous molecules, such as glutathione 183,184,185,186,187, or the expression of enzymes like superoxide dismutases (SOD1 and SOD2), catalase, and peroxidase 188. About 1% of ROS escape elimination and can be released to the cytosol by crossing the OMM, where they can attack and modify DNA, lipids and proteins affecting cell survival 189.

VDAC1 has been proposed to mediate ROS release from the IMS to the cytosol. This is based on the finding that ROS release from mitochondria was decreased when HK-I and HK-II bound to VDAC1 were over-expressed in HEK cells, reducing intracellular ROS levels 190,191,192 and protecting against oxidant-induced cell death 190,193. In tumor cells, VDAC opening or closing increases or decreases OXPHOS and subsequently increases or decreases ROS generation, respectively. Erastin, a small molecule that antagonizes the inhibitory effect of tubulin on VDAC, increases mitochondrial ΔΨ, NADH and ROS production 85. Thus, blockage of tubulin-dependent VDAC inhibition works as a pro-oxidant anti-Warburg metabolic switch to promote cancer cell death 88,194. By contrast, VDAC1 closure by DIDS and dextran sulfate inhibits the efflux of O_2_-• to the cytosol and increases the steady-state level of O_2_•-, sensitizing mitochondria to Ca^2+^-induced MPT 195.

Cysteine residues, often involved in redox reactions, metal coordination and thiol-disulfide interchanges, are extremely vulnerable to oxidation by ROS. VDAC has been proposed to function in physiological redox regulation via the modification of the sulfhydryl groups of VDAC 196. In humans, VDAC1 contains two cysteines, VDAC2 contains nine cysteines and VDAC3 contains six cysteines, all of which are predicted to protrude towards the IMS and can be subjected to oxidation by ROS 4. We have shown that for VDAC1, deletion of both cysteines does not affect channel conductance, VDAC1 oligomerization or apoptosis 53. Cysteines contribute to the folding, function and stability of hVDAC2. VDAC3 was found to be the target of mitochondrial ROS specifically generated by complex III and was proposed to act as a sensor of the oxidative state of the IMS via cysteine residue modification 197.

Accumulating evidence indicates that ROS play a key role in Cyto *c* release from mitochondria and that this involves VDAC1. Apoptosis-inducing agents, such as inorganic arsenic compounds 198,199 and doxorubicin 200, induce apoptosis by inducing ROS generation. The inhibition of O_2_-•-induced apoptosis by DIDS, an inhibitor of VDAC channel activity, or by anti-VDAC1 antibodies 12,129,201, suggests that O_2_-• induces Cyto *c* release via VDAC1-dependent permeabilization of the OMM 129. Moreover, O_2_-• was found to evoke Cyto *c* release in VDAC1-reconstituted liposomes 129. In other studies, it was found that ROS-induced alterations of VDAC1 and/or ANT could make the PTP selective for Cyto *c* release, without causing further mitochondrial damage 129,202. Moreover, it was shown that ROS induced up-regulation of VDAC1 that could be prevented by the ROS chelator, epigallocatechin 144. It has been suggested that ROS-mediated Cyto *c* and SOD1 release from mitochondria involves VDAC, leading to increased susceptibility of mitochondria to oxidative stress and apoptosis 203.

VDAC is also affected by hypoxic conditions shown to induce cleavage at the C-terminal end of the protein (VDAC1-ΔC), with such cleavage being prevented upon silencing of HIF-1α expression 204,205. It was proposed that hypoxia, by inducing formation of VDAC1-ΔC, confers selective protection from apoptosis that allows maintenance of ATP and cell survival in hypoxia 206.

## VDAC1 AS A HUB PROTEIN - MODULATION OF VDAC1-MEDIATED APOPTOSIS AND METABOLISM VIA INTERACTING PROTEINS 

As presented above, VDAC1 is crucial for many cellular processes, including metabolism, Ca^2+^ homeostasis, apoptosis, and other activities regulated via the interaction of VDAC1 with many proteins associated with cell survival and cellular death pathways 1,29,30,31. Indeed, VDAC1 is considered as a hub protein, interacting with over 100 proteins that regulate the integration of mitochondrial functions with other cellular activities 207. VDAC1 serves as an anchor protein for diverse sets of cytosolic, ER, and mitochondrial proteins 12,208 that together regulate metabolic and signaling pathways, provide energy for cellular functions, or trigger cell death. Thus, VDAC1 appears to be a convergence point for a variety of cell survival and death signals, mediated via association with ligands and proteins.

In support of this viewpoint, the conserved nature of VDAC1 1 is in agreement with the finding that hub proteins are more evolutionarily conserved than are non-hub proteins 209. VDAC1 protein-protein interaction (PPI) networks contain both hub-bottlenecks 210 (namely nodes with high degree values constituting vulnerable areas of the network) and/or bottlenecks (those with high "betweenness" centrality scores, corresponding to key intersecting nodes 211). The VDAC1 interactome includes proteins involved in metabolism, apoptosis, signal transduction, anti-oxidation, and DNA- and RNA-associated proteins and more (Supplemental Table S1) 1,29,31,32. Furthermore, these proteins may be located in the OMM, IMM, the IMS, the cytosol, ER, plasma membrane, and nucleus. Importantly, we have been able to develop VDAC1-based peptides which can interfere with these interactions, leading to impaired cell metabolism and apoptosis 25,26,27,212,213.

### Interactions of VDAC1 with metabolism-related proteins

VDAC1 displays binding sites for a large number of metabolism-related proteins, such as glycerol kinase (GK), HK, c-Raf kinase, ANT, tubulin, 1,29,30,31 and the glycolytic enzyme GAPDH (glyceraldehyde 3-phosphate dehydrogenase) 214. Mitochondrial creatine kinase (MtCK), in its octameric state, interacts with VDAC1 35 and causes decreased affinity of VDAC1 for HK and Bax 215 (Supplemental Table S1). HK binding to VDAC1 25,104,118,122,124,216 allows direct coupling of mitochondrially generated ATP to glucose phosphorylation. Thus, the formation of a VDAC1-HK complex coordinates glycolytic flux with the actions of the TCA cycle and ATP synthase 1,33,81.

The OMM protein CPT1a that catalyzes the primary step of fatty acid oxidation interacts with VDAC1 65. Another OMM protein interacting with VDAC1 is the TSPO, involved in the transport of cholesterol into mitochondria 217. Aldolase, involved in gluconeogenesis and glycolysis, was also shown to interact with VDAC1 17.

### Interactions of VDAC1 with apoptosis-related proteins

The Bcl-2 family comprises pro-apoptotic (e.g. Bid, Bax, Bim and Bak) and anti-apoptotic (e.g. Bcl-2 and Bcl-xL) members that up- or down-regulate apoptosis, respectively 218,219. VDAC1 function in apoptosis can be regulated by interactions with anti-apoptotic proteins, such as Bcl2 and Bcl-xL 23,25,26,117,220,221,222,223, resulting in inhibition of apoptotic pathways. Bcl-2 and Bcl-xL were shown to interact with bilayer-reconstituted VDAC1 and subsequently to reduce the channel conductance of native but not mutated VDAC1, as well as to protect against apoptosis in cells expressing native but not mutated VDAC1 25,26. The VDAC1 domains that interact with Bcl-2 and Bcl-xL to confer anti-apoptotic activity were identified by site-directed mutagenesis 25. Mcl-1 has been shown to directly interact with VDAC to increase mitochondrial Ca^2+^ uptake and ROS generation 224. The HK-VDAC interaction also prevents release of pro-apoptotic factors, such as Cyto *c*, and subsequent apoptosis. Thus, HK plays a role in tumor cell survival via inhibition of apoptosis 118. The interaction between TSPO and VDAC is considered to play a role in the activation of the mitochondrial apoptosis pathway, given the reported grouping of TSPO molecules around VDAC, potentially reflecting TSPO polymerization 225, and the increased ROS generation by TSPO in the proximity of VDAC, leading to apoptosis induction 225,226. Nek1 (NIMA-related protein kinase 1) phosphorylates VDAC1 on serine 193, with this leading to apoptosis inhibition 146,227. Finally, the pro-apoptotic protein BNIP3 was shown to interact with VDAC so as to induce mitochondrial release of endonuclease G 228.

### Interactions of VDAC1 with cytoskeletal proteins 

VDAC1 interacts with several cytoskeletal proteins, such as gelsolin (Gsn), with this interaction resulting in inhibited VDAC1 channel activity and Cyto *c* release from liposomes through direct binding to VDAC1 in a Ca^2+^-dependent manner 229,230. Tubulin was shown to associate with VDAC1 231 and induce VDAC1 closure 86, proposed to sustain the Warburg effect 232. It was further proposed that tubulin, VDAC1, and MtCK form a super-complex that is structurally and functionally coupled to the ATP synthasome 233. G-actin directly and selectively binds to VDAC in yeast, 234, reducing conductance of the *Neurospora crassa* VDAC channel 235. Microtubule-associated protein 2 (MAP2) was shown to bind VDAC 236. The interaction of VDAC1 with Tctex-1/DYNLT1 (dynein light chain) was also demonstrated 237.

### Interactions of VDAC1 with signaling proteins 

Superoxide dismutase 1 (SOD1) is a predominantly cytosolic protein, with mutant SOD1 being present mostly in fractions enriched for mitochondria 238,239,240. Mutant SOD1 associated with amyotrophic lateral sclerosis (ALS) bound to bilayer-reconstituted VDAC1 and inhibited its channel conductance 241. Mutant SOD1 also interacted with Bcl2 protein and altered the interaction between Bcl-2 and VDAC1, thus reducing OMM permeability 242.

Endothelial NO synthase (eNOS) was also found to bind VDAC1. Such interactions amplified eNOS activity in an intracellular Ca^2+^-mediated manner 243. These findings suggest that the interaction between VDAC and eNOS may be important for regulating eNOS activity and modulation of VDAC 243.

The mitochondrial anti-viral signaling protein MAVS, also known as IPS-1, VISA, or Cardif 244, and localized in the OMM, was demonstrated to mediate its pro-apoptotic activity via VDAC1 and to modulate VDAC1 protein stability via the ubiquitin-proteasome pathway 245. VDAC was further proposed to interact with the L-type Ca^2+^ channel 246.

Several additional proteins were shown or proposed to directly interact with VDAC1 (Supplemental Table S1). These include PBP74, also known as mtHSP70/GRP75/mortalin 237, CRYAB (α-crystallin B) 247 and α-synuclein 248. VDAC1-interacting protein complexes mediate and/or regulate metabolic, apoptotic, and other processes that may be impaired in disease.

## VDAC INVOLVEMENT IN DISEASE 

Mitochondria occupy a central position in cell life and death and mitochondrial dysfunction has been implicated in many diseases, including cancer, Alzheimer’s disease (AD), Parkinson's disease (PD), amyotrophic lateral sclerosis (ALS), diabetes, and cardiovascular diseases (CVDs). VDAC1 functions as mitochondria gatekeeper that regulates ATP production, Ca^2+^ homeostasis and apoptosis execution, all indispensable for proper mitochondrial function, and consequently, for cell normal physiology. Thus, the association of VDAC with various diseases is not surprising. Furthermore, VDAC over-expression is a common feature of cancer, AD, type 2 diabetes (T2D) and CVDs. The over-expression of VDAC1 in cancer 54,59, in affected regions of AD brains 97,98,99, in β-cells of T2D 101 and in CVDs 250, is a feature common to these diseases. As VDAC1 over-expression induces apoptotic cell death 58,113,119,120,122, its over-expression in CVDs, AD and T2D, may be a common mechanism in these pathologies.

### The cancer-mitochondria-metabolism-apoptosis-VDAC1 link 

Cancer is a complex disease in which cells acquire a common set of properties, including unlimited proliferation, metabolic reprograming, and resistance to anti-proliferative and apoptotic cues 78,250. Emerging evidence indicates that metabolic reprogramming, which supports macromolecule synthesis, bioenergetics demands, and cellular survival is a characteristic of nearly all cancers 68,251. Over the years, Otto Warburg's view of cancer as a metabolic disease was gradually displaced with the view of cancer as a genetic disease 252. Today, however, cancer is again being seen as a metabolic disease, primarily associated with impaired mitochondrial function and metabolism 75,79.

VDAC1 is highly expressed in different tumors 54,59, contributing to their metabolism via the transport of various metabolites, and the binding and channeling of mitochondrial ATP directly to HK 5. This results in mitochondria regulating glycolytic flux with that actions of the TCA cycle and ATP synthase to fulfill the requirements of tumors for metabolites or metabolite precursors. Indeed, tumors switch their HK expression pattern predominantly to present the VDAC1-binding isoforms (HK-I, HK-II) 253. The requirement of cancer cells for VDAC1 is demonstrated by down-regulation of VDAC1, resulting in reduced metabolite exchange between mitochondria and cytosol and inhibition of cell and tumor growth 32,58,59,90,91.

VDAC1 also regulates apoptosis in cancer cells by interacting with the anti-apoptotic proteins Bcl-2 and Bcl-xL 23,25,26,254 and HK 23,27, interactions that protect tumor cells from cell death 23,27. Thus, activating mitochondria-mediated apoptosis directly or via generating stress responses 255,256,257 is a strategy to treat cancer. Indeed, a large number of anti-cancer chemotherapeutic agents exert their therapeutic action by inducing apoptosis of malignant cells 258,259,260,261,262,263, mainly by activating the Cyto *c*/caspase-9 pathway. These include etoposide, doxorubicin, lonidamine, betulinic acid, arsenite, CD437, and several amphiphilic cationic α-helical peptides 264. Therefore, targeted activation of apoptosis in cancerous tissues may be exploited as a potential route to cancer therapy 265. However, they do not act on cancer stem cells (CSCs), which are resistant to chemo- and radio-therapies 266,267,268. The CSC hypothesis postulates that a sub-population of malignant cells constantly supply the tumor with cancerous cells. CSCs, as embryonic and somatic stem cells, have self-renewal and multi-potent differentiation abilities 269,270. Recent studies from the Shoshan-Barmatz group 91,213 have demonstrated novel strategies for eliminating CSCs.

Regulation of VDAC1 expression by miRNA was demonstrated in several studies. In serum-starved cervical cancer cells, miR320a promoted mitophagy 93, while ectopic over-expression of miR320a blocked tumor cell proliferation and invasion in NSCLC, both *in vitro* and in *vivo* 271. Another miRNA species, miR-7, was shown to inhibit VDAC1 expression, proliferation and metastasis in hepatocellular carcinoma 94, possibly by affecting the PTP 95.

Thus, the importance of VDAC1 for cancer cell survival is clearly reflected in the above findings, with silencing VDAC1 expression in cancer cells resulting in a multi-pronged attack on cancer hallmarks.

### Neurodegenerative diseases, mitochondria, apoptosis, and VDAC 

There is emerging evidence connecting mitochondrial dysfunction to neurodegenerative disorders 169. In PD, Huntington’s disease (HD), ALS and AD, impaired mitochondrial function has been reported 272, with a focus on the involvement of mitochondria-mediated apoptotic death 273. Mitochondrial dysfunction was proposed as an early event in AD pathogenesis, as reflected in reduced metabolism, increased ROS, lipid peroxidation and disruption of Ca^2+^-homeostasis, 274,275,276. Moreover, mitochondria-mediated apoptosis is common to neurological disorders in which premature neuron death is implicated 273,277,278, with caspases playing dominant roles 279,280,281. Amyloid beta (Aβ) also affects mitochondrial respiration 282 and activates Cyto *c* release, thereby promoting apoptosis 283.

Several studies suggested that VDAC malfunction is associated with AD 284,285,286,287, Down's syndrome 287, and familial ALS 241,288. High levels of VDAC1 were demonstrated in the dystrophic neurites of Aβ deposits in post-mortem brains of AD patients and in amyloid precursor protein (APP) transgenic mice, where Aβ-VDAC interactions are toxic to AD-affected neurons 97,98,289,287,289,290,291. The expression of hVDAC-2 was shown to be associated with neurodegenerative diseases, including ALS 288, epilepsy 292, and AD 286.

As VDAC1 over-expression was shown to lead to apoptotic cell death 58,113,119,120,122 and high-levels of VDAC1 were found in the dystrophic neurites of Aβ deposits in AD post-mortem brains and APP transgenic mice 97,98,99, we propose that over-expressed VDAC is associated with neuronal cell death 291.

We have demonstrated that Aβ interacts directly with VDAC1, specifically with the VDAC1 N-terminal region and that VDAC1 is required for Aβ entry into the cell, as well as for Aβ-mediated apoptosis, with Aβ cell penetration and toxicity being prevented in cells depleted of VDAC1 by siRNA 291. VDAC was also shown to interact with phosphorylated Tau, leading to mitochondrial dysfunction 290. In addition, an increase in nitrated VDAC1 levels in AD was reported, reflecting oxidative damage to VDAC 293, and possibly affecting cell energy and metabolite homeostasis 284. The involvement of plasmalemmal VDAC in AD was also proposed 285,289.

The relationship between VDAC1 expression levels and neurodegenerative disorders is also reflected in the finding that in patients and animal models of several neurodegenerative disorders, such as AD, HD, and spinocerebellar ataxias, miR-29a expression levels were reduced 294. miR-29a was also shown to regulate cell survival of astrocytes differentially by targeting VDAC1 295. These findings suggest that VDAC1 down-regulation by miR-29 is an important aspect of neuronal cell survival in the brain 294. As VDAC1 over-expression triggers apoptosis 120,121,122, and high-levels of VDAC1 were demonstrated in AD post-mortem brains and in AD-like transgenic mice 99, the reported decrease in miR-29a in AD 294 may be associated with neuronal cell death. Indeed, miR-320a-mediated down-regulation of VDAC1 expression has been proposed as a novel therapeutic target for astroglia-mediated HIV-1 neuropathogenesis 296.

Finally, several proteins interacting with VDAC, such as SOD1, α-synuclein and ApoE, were proposed to be involved in several neurodegenerative diseases, affecting intraneuronal Ca^2+^ 155. These findings point to VDAC1 as a potential target for novel therapeutic strategies for neurodegenerative diseases.

### T2D, metabolism, mitochondria and VDAC1

T2D is the most common metabolic disease 297. Defective insulin secretion, insulin resistance at target tissues and a loss of functional β-cells contribute to T2D, and dysregulation of glucose homeostasis 298. Recently, it has been shown that hyperglycemia increases VDAC1 expression in pancreatic β-cells 101 and in the kidney 102. VDAC1 levels were increased in mouse coronary vascular endothelial cells (MCECs) isolated from diabetic mice. This was associated with increased [Ca^2+^]m, O_2_ production, and PTP opening activity 299. Down-regulation of VDAC1 in diabetic MCECs decreased [Ca^2+^]m and subsequently affected PTP activity and ROS production 300. As glucose-stimulated insulin secretion depends on the generation of ATP and other metabolites in the mitochondria 301, and VDAC1 regulates energy and metabolism, VDAC1 is thus required for insulin secretion. Recently, lncRNA-H19/miR-675 was reported to regulate high glucose-induced apoptosis by targeting VDAC1, and thus provides a novel therapeutic strategy for the treatment of diabetic cardiomyopathy 96. These findings point to the connection between VDAC, mitochondrial function and the pathogenesis of T2D.

### Cardiovascular diseases, mitochondria, apoptosis and VDAC 

It is known that most CVDs evolve into heart failure and that the loss of cardiac myocytes plays a critical role in the pathogenesis of CVDs. Activation of mitochondria-mediated apoptosis has been implicated in ischemia/reperfusion injury 302. VDAC levels were increased in cardiomyoblast H9c2 cells 249. As VDAC1 over-expression is associated with apoptosis 58,113,119,120,122, it is possible that increased cardiomyocyte susceptibility to mitochondrial-mediated cell death is related to the increase in VDAC1 levels. Indeed, the effect of resveratrol against myocardial ischemia/reperfusion injury showed involvement of VDAC1 down-regulation 100.

The findings presented above suggest that modulating VDAC1 expression levels or its apoptotic activity are possible strategies to either activate apoptosis in cancer or inhibit apoptosis in CVDs, AD and T2D.

## UNRAVELING VDAC1-BASED THERAPIES 

VDAC1-based strategies are expected to be effective in various diseases characterized by altered cell metabolism and/or apoptosis and by VDAC1 over-expression. As VDAC1 over-expression induces apoptotic cell death 58,113,119,120,122, we suggest that this may be a common mechanism in the pathology of CVDs, AD and T2D. Modulating VDAC1 expression levels or its apoptotic activity are possible strategies to either activate apoptosis in cancer or inhibit apoptosis in CVDs, AD and T2D. In this review, VDAC1-based therapeutic strategies targeting tumor cells are presented. These cancer therapy strategies include siRNA altering the normal functioning of cancer cells, leading to growth arrest, and VDAC1-based peptides that impair energy homeostasis and minimize the self-defense mechanisms of these cells, and that can be used to overcome protective and pro-survival actions taken by cancer cells.

### VDAC1-depletion using RNAi

Specifically targeting metabolism in cancer cells presents a potential therapeutic strategy. However, although glucose metabolism is increased in cancer cells, these cells mostly use the same glycolytic enzymes as do normal cells, so that the choice of glycolytic enzymes as targets for cancer treatment may increase the risk of adverse and undesirable consequences 303. Targeting VDAC1, acting as a ‘governor’ of mitochondrial function, regulating cellular energy and metabolism, and over-expressed in cancer, offers a unique target for anti-cancer therapies. Down-regulation of VDAC1 addresses the cancer trademark of cell metabolic and energy reprogramming, leading to disrupted cancer cell energy and metabolism homeostasis.

VDAC1 depletion using specific siRNA (si-VDAC1) led to reduced cellular ATP levels and inhibited cell and tumor growth in cervical and lung cancers 58,59,90. Using a sub-cutaneous and intracranial-orthotopic GBM model, we found that VDAC1 depletion resulted in inhibited tumor growth, with the residual tumor showing reversed oncogenic properties, including metabolic reprograming and inhibited proliferation, angiogenesis, EMT, invasiveness and stemness, leading to differentiation into neuron- and astrocyte-like cells 91 (**Fig. 4**).

### VDAC1-based peptides as potential anti-cancer therapy

A hallmark of cancer cells is their ability to suppress pro-apoptotic pathways and/or to activate anti-apoptotic mechanisms 78,250 associated with drug resistance 304, such as the Bcl-2 family of proteins and HK, preventing the release of Cyto *c* from mitochondria. Since the anti-apoptotic proteins HK-I, HK-II, Bcl2 and Bcl-xL have been found to be expressed at high levels in many types of cancer 81,253,305,306,307,308,309,310 and interact with VDAC1 1,22,23,24,25,26,27,81,82,84,118,122,124,128,220,311,312, the interaction of VDAC1with these proteins is proposed as an appropriate target to induce apoptosis.

We have engineered VDAC1-based peptides that interfere with the activity of the pro-survival proteins Bcl-2, Bcl-xL and HK 23,25,26,27,118,122,212. Via point mutations, VDAC1 domains and amino acid residues important for interactions with HK, Bcl-2 and Bcl-xL were identified and cell-penetrating VDAC1-based peptides targeting these interactions were designed and tested 23,25,26,27,118,122. These VDAC1-based peptides were found to induce cancer cell death in a panel of genetically characterized cancer cell lines, regardless of cancer type or mutation status, with perceived specificity toward cancerous cells 23,27,212. Studies demonstrated a triple mode of action, namely energy and metabolism impairment, interference with the action of anti-apoptotic proteins, and a triggering of cell death.

In an *ex vivo* study, cell-penetrating VDAC1-based peptides were found to induce apoptotic cell death in the cancerous B-cells of peripheral blood mononuclear cells obtained from chronic lymphocytic leukemia (CLL) patients, yet spared those obtained from healthy donors, pointing to the potential of VDAC1-based peptides as an innovative and effective anti-CLL therapy.

In a GBM mouse model, i.v.-administered VDAC1-based peptide Tf-D-LP4 crossed the blood-brain barrier and was found to inhibit tumor growth by inducing apoptosis and over-expression of apoptotic proteins 213. Such treatment simultaneously attacked several cancer hallmarks, causing impairment of energy and metabolic homeostasis, inhibition of tumor growth and induction of apoptosis. VDAC1-based peptides, expecting to also affect other cancers, provide the opportunity for the development of new anti-cancer therapies that will allow overcoming the chemo-resistance of cancer cells.

In summary, VDAC1 functions in ATP production and metabolism, Ca^2+^ homeostasis and apoptosis execution are indispensable for proper mitochondrial function of cancer cell, and consequently, for cell activity. These VDAC-mediated activities are regulated via interactions of VDAC1 with many proteins that are critically involved in the regulation of cell survival and cellular death pathways. VDAC1, standing at the crossroads between mitochondrial-mediated energy and metabolism and apoptosis, is a potential target for treating cancer and other diseases involving dysregulated metabolism and/or apoptosis and where VDAC1 is over-expressed. Thus, a new generation of VDAC1-based therapeutics may impact the treatment of a variety of diseases.

To conclude, the dysregulated cell stress response involves mitochondria dysfunction and this play a critical role in tumorigenesis, and other disease, such as Alzheimer’s disease, some cardiovascular disease and type 2 diabetes. The role of VDAC1 in Ca^2+^ homeostasis, energy production and oxidative stress, and with VDAC1 serving as a hub protein interacting with over 100 proteins allow it to mediate and regulate the integration of mitochondrial functions with cellular activities. Thus, VDAC1, standing at the crossroads between mitochondrial metabolite transport, apoptosis and other cell stress-associated processes, serves as the mitochondrial gatekeeper. This, together with its over-expression in cancer and other diseases, including Alzheimer’s disease, some cardiovascular diseases and type 2 diabetes, involves VDAC1 in the cell stress response and thus represents a target to modulate the biology of cancer and other diseases.

## SUPPLEMENTAL MATERIAL

Click here for supplemental data file.

All supplemental data for this article are also available online at http://www.cell-stress.com/researcharticles/vdac1-at-the-crossroads-of-cell-metabolism-apoptosis-and-cell-stress/.

## Abbreviations

Aβ: Amyloid beta
AD: Alzheimer’s disease
ALS: amyotrophic lateral sclerosis
AIF: apoptosis-inducing factor
ANT: adenine nucleotide translocase
Bcl-2: B-cell lymphoma 2
CSC: cancer stem cell
CVD: cardiovascular disease
Cyto c: cytochrome c
DIDS: 4,4-diisothiocyanostilbene-2,2-disulfonic acid
HK: hexokinase
IMM: inner mitochondrial membrane
IMS: intermembrane space
NSCLC: non-small cell lung cancer
miRNA: micro RNA
OMM: outer mitochondrial membrane
OXPHOS: oxidative phosphorylation
PTP: permeability transition pore
RNAi: RNA interference
ROS: reactive oxygen species
TSPO: translocator protein
T2D: type 2 diabetes
VDAC: voltage-dependent anion channel
