# Supplementary material for: VDAC1 at the crossroads of cell metabolism, apoptosis and cell stress
Source: Cell Stress. 2017 Oct 1;1(1):11–36. doi: 10.15698/cst2017.10.104 (PMC6287957; doi:10.15698/cst2017.10.104)
Supplement: Supplementary file 1 [file ces-01-011-s01.pdf]

Supplemental Table S1: VDAC1-interacting proteins

Protein	Function	Cellular localization	Interaction assay	Reference
**Apoptosis**				
**AIF1**-Apoptosis-inducing factor 1	Apoptosis	Mitochondria	Affinity Capture-MS	[1]
**Bak**-Bcl-2 homologous antagonist/killer	Apoptosis	Mitochondria	Affinity Capture-Western	[2, 3, 4]
**Bax**-Bcl-2-associated X protein	Apoptosis	Mitochondria	Reconstituted Complex	[5, 6, 7]
**Bcl2**- B-cell lymphoma 2	Apoptosis inhibitor	Mitochondria	Affinity Capture-Western	[8, 9, 10, 11]
**Bcl-xL**- B-cell lymphoma-extra large	Apoptosis inhibitor	Mitochondria	Western, Reconstituted Complex	[4, 5, 6, 12, 13, 14,15]
**Bim**- Bcl-2-like protein 11	Apoptosis	Mitochondria	Affinity Capture-Western	[16, 17]
**BNIP3**-BCL2/adenovirus E1B 19 kDa protein-interacting protein 3	Apoptosis	Mitochondria	Biochemical Activity	[18]
**Bop**- C22orf29	Apoptosis	Mitochondria	Affinity Capture-Western	[19]
**IκBα**- NF-kappa-B inhibitor alpha	Apoptosis inhibitor	Cytosol, Mitochondria	Affinity Capture-Western	[20]
**MCL1**- Induced myeloid leukemia differentiation protein	Apoptosis	Mitochondria	Reconstituted Complex	[6]
**tBid**-BH3-interacting domain death agonist	Apoptosis	Mitochondria	Reconstituted Complex	[21]
**Apoptosis & metabolism**				
**Bag3**- Bcl2-associated athanogene 3	Metabolism/Apoptosis	Mitochondria	Affinity Capture-MS	[22]
**HK-1**-Hexokinase-1	Metabolism/Apoptosis inhibitor	Cytosol	Affinity Capture-Western	[8, 9, 23, 24, 25]
**HK-2**-Hexokinase-2	Metabolism/Apoptosis inhibitor	Cytosol	Affinity Capture-Western	[8, 9, 23]
**Metabolism & energy**				
**ALDOA**-Fructose-biphosphate aldolase A	Metabolism/scaffolding protein	Cytosol	Reconstituted Complex	[26]
**ANT**-ATP/ADP translocase	Channel	Mitochondria	Affinity Capture-Western	[27, 28]
**CPT1a**-Carnitine O-palmitoyltransferase 1	Metabolism	Mitochondria	Affinity Capture-Western	[29]
**Cytochrome *c***	Metabolism/Apoptosis	Mitochondria	Reconstituted Complex	[30]
**GAPDH**-Glyceraldehyde-3-phosphate dehydrogenase	Metabolism	Cytosol	Reconstituted Complex	[31]
**GK**- Glycerol kinase	Metabolism/Glycerol		Affinity Capture-Western	[32]
**HAL**- Histidine ammonia-lyase	Metabolism/L-histidine degradation	Cytosol	Affinity Capture-MS	[33]
**CKMT1A**-Creatine kinase U-type	Metabolism	Mitochondria	Reconstituted Complex, SRP	[34, 35]
**StAR**-Steroidogenic acute regulatory proteins	Metabolism/cholesterol transport	Mitochondria	Affinity Capture-Western	[36]
**TSPO**-Translocator protein	Metabolism/cholesterol transport	Mitochondria	Affinity Capture-Western	[37, 38]
**Transporters**				
**CYPD**-Cyclophilin D	IMM permeability	Mitochondria	Affinity Capture-Western	[39]
**GLT-1** (Slc1a2)-Excitatory amino acid transporter 2	Glutamate transporter	Plasma membrane	Affinity Capture-Western	[40]
**MMGT1**-Membrane magnesium transporter 1	Mg^{2+} transport	ER-Golgi	Affinity Capture-MS	[1]

**NXF1**- Nuclear RNA export factor 1	Export of mRNA	Nucleus, cytosol	Affinity Capture-RNA	[41]
**PLIN3**-Perilipin-3	Transport of mannose 6 phosphate receptor to Golgi	Cytoplasm, Endosome membrane	Affinity Capture-RNA	[33]
		**Mitochondria**		
**ICT1**- Peptidyl-tRNA hydrolase	Peptidyl-tRNA hydrolase component	Mitochondria	Affinity Capture-MS	[42]
**MAVS**-Mitochondrial antiviral-signaling protein	Intracellular immune defense	Mitochondria, peroxisome	Affinity Capture-Western	[43]
**MTX2**- Metaxin-2	Transport	Mitochondria	Affinity Capture-MS	[1]
**RMDN3**- Regulator of microtubule dynamics protein 3	Calcium homeostasis	Mitochondria outer membrane, Cytosol	Affinity Capture-MS	[1]
**SPG7**-Paraplegin	Protease	Mitochondria	Affinity Capture-Western	[44]
**TOMM20**-Import receptor subunit TOM20 homolog	Mitochondrial receptor	Mitochondria	Reconstituted Complex	[45]
		**Redox potential**		
**eNOS**-Nitric oxide synthase, endothelial	NO production	Cytosol, Golgi, plasma membrane	Affinity Capture-Western	[46]
**SOD1**-Superoxide dismutase	Antioxidant	Cytosol, nucleus mitochondria	Affinity Capture-Western	[47]
**TXNL1**-Thioredoxin-like protein 1	Redox reaction	Cytosol	Two-hybrid	[48]
		**ER & Golgi-associated**		
**GABAR-APL2**- GABAR-associated protein-like 2	Transport	ER-Golgi	Affinity Capture-MS	[49]
**GOLT1B**- Vesicle transport protein GOT1B	Glycosaminoglycan metabolism/transport	ER-Golgi	Affinity Capture-MS	[1]
**GRP78**-78 kDa glucose-regulated protein	Protein folding	ER	Two-hybrid	[48]
**PSENEN**-γ-secretase subunit PEN-2	Endoprotease	ER-Golgi	Affinity Capture-MS	[50]
		**Kinases**		
**CDK2**-Cyclin-dependent kinase 2	Kinase	Cytosol	Affinity Capture-MS	[51]
**CSNK2B**-Casein kinase II subunit β	Kinase	Cytosol, Nucleus	Two-hybrid	[52]
**MAPK3** (ERK1)-Mitogen-activated kinase 3	Kinase	Cytosol	Affinity Capture-MS, FRET	[53]
**MPS1**- Dual specificity protein kinase TTK	Kinase	Cytosol	Affinity Capture-Western	[54]
**NEK1**-Serine/threonine-protein kinase	Kinase	Cytosol, Nucleus	Biochemical Activity	[55, 56]
**PANK2**-Pantothenate kinase 2	Kinase/CoA biosynthesis	Mitochondria	Affinity Capture-MS	[49]
**PRKCE**- Protein kinase C ε type	Kinase	Cytosol	Western, Reconstituted Complex	[57]
**c-RAF**-Proto-oncogene serine/threonine-protein kinase	Kinase	Cytosol	Affinity Capture-Western	[58]
		**Cell receptors**		
**ADRB2**- β-2 adrenergic receptor	Receptor	Plasma Membrane	Protein fragment complementation assay	[59]
**APOE**-Apoliprotein E	Co-Receptor	Plasma Membrane	Affinity Capture-MS	[35]
**APP**- Amyloid beta A4 protein	Receptor	Plasma Membrane	Affinity Capture-Western	[60]

**CD4**-T-cell surface glycoprotein	Receptor	Plasma Membrane	Affinity Capture-MS	[61]
**FGFR1OP2**- FGFR1 oncogene partner 2	Receptor/kinase	Plasma Membrane	Affinity Capture-MS	[1]
**IP3R**- Inositol trisphosphate receptor	Ca^{2+} release channel	ER	Two-hybrid	[48, 62]
**VCAM1**-Vascular cell adhesion protein 1		Membrane	Affinity Capture-MS	[63]
**Vesicle proteins**				
**HTT**- Huntingtin	Transport, vesicle activity	Cytosol, Nucleus	Affinity Capture-MS	[1]
**RAB2A, RAB5C, RAB7A**- Ras-related protein 2A, 5C, 7A	Endo-lysosomal trafficking	Cytoplasmic vesicles	Affinity Capture-MS	[1]
**TMED2**- Transmembrane emp24 domain-containing protein 2	Receptor, vesicle coat formation	ER-Golgi, Cytosol	Affinity Capture-MS	[1]
**VAPA**-Vesicle-associated membrane protein-associated protein A	Vesical	Membrane	Affinity Capture-MS, Co-fractionation	[1, 64, 65]
**Structural**				
**ACTG**- Actin, cytoplasmic 2	Cell motility	Cytosol	SPR	[66, 67]
**CAV1**-Caveolin-1	Cytoskeletal		Affinity Capture-Western	[68]
**DYNLT1**-Dynein light chain Tctex-type 1	Motor protein	Cytosol	Two-hybrid, Reconstituted Complex	[48, 69]
**FLNB**-Filamin-B	Cytoskeletal	Cytosol	Two-hybrid	[48]
**FMNL1**- Formin-like protein1	Cytoskeletal	Cytosol	Affinity Capture-MS	[70]
**GSN**-Gelsolin	Cytoskeletal	Cytosol	Affinity Capture-MS	[71]
**HAUS3**-Augmin-like complex subunit 3	Cytoskeletal	Cytosol	Affinity Capture-MS	[72]
**KIF5B**- Kinesin-1 heavy chain	Microtubule-associated		Affinity Capture-MS	[49]
**MAP2**-Microtubule-associated protein 2	Cytoskeletal	Cytosol	Affinity Capture-MS	[73]
**MYO19**- Unconventional myosin-XIX	Cytoskeletal	Cytosol	Affinity Capture-MS	[1]
**SCIN**-Adseverin	Cytoskeletal	Cytosol	Two-hybrid	[74]
**TUBA1B**-Tubulin alpha-1B chain	Cytoskeletal	Cytosol	Affinity Capture-Western, PLB	[75, 76, 77]
**Ubiquitination**				
**ASB14, ASB17**-Ankyrin repeat and SOCS box protein 14, 17	Ubiquitination	Cytosol, Nucleus	Affinity Capture-MS	[78]
**FOXRED2**- FAD-dependent oxidoreductase domain-containing protein 2	Ubiquitination	ER	Affinity Capture-MS	[79]
**PARK2**- E3 ubiquitin-protein ligase Parkin	Ubiquitination	Cytosol, Mitochondria		[80-83]
**UBAC2**- Ubiquitin-associated domain-containing protein 2	Restricts protein trafficking	ER	Affinity Capture-MS	[79]
**UBC**-Ubiquitin C	Ubiquitination	Cytosol, Nucleus	Affinity Capture-MS	[84-87]
**DNA-associated**				
**ATF2**- Cyclic AMP-dependent transcription factor	Transcriptional activator	Cytosol, nucleus, mitochondria	Affinity Capture-Western	[24]
**HDAC5**-Histone deacetylase 5	Histone deacetylase	Nucleus	Affinity Capture-MS	[88]

**MCC**- Colorectal mutant cancer protein	Inhibits DNA binding of β-catenin/ TCF/ LEF TFs	Nucleus	Affinity Capture-MS	[49]
**MDC1**- Mediator of DNA damage checkpoint protein 1	DNA damage check point protein	Nucleus	Affinity Capture-MS	[89]
**MED20**- Mediator of RNA polymerase II transcription subunit 20	RNA polymerase II transcription mediator	Nucleus	Affinity Capture-MS	[90]
**SIRT7**-NAD-dependent protein deacetylase sirtuin-7	Deacetylase	Nucleus	Affinity Capture-MS	[91]
**SPRTN**- SprT-like domain-containing protein Spartan	Regulator of UV-induced DNA damage	Cytosol, nucleus	Affinity Capture-MS	[92]
**ZNF622**-Zinc finger protein 622	Transcription activation	Nucleus, cytosol	Two-hybrid	[48]
		**RNA-associated**		
**AI837181**	Poly(A) RNA binding		Affinity Capture-MS	[72, 93]
**EWSR1**- RNA-binding protein EWS	Transcriptional repressor	Cytosol, nucleus, membrane	Proximity labeled-MS	[93]
**MOV10**- Putative helicase	RNA helicase	Cytosol	Affinity Capture-MS	[41]
**NTRK1**- High affinity nerve growth factor receptor	Receptor tyrosine kinase	Membrane	Affinity Capture-MS	[94]
		**Chaperons**		
**CRYAB**- α-crystallin B	Chaperone-like activity	Cytosol, nucleus	Affinity Capture-MS	[95]
**DNAJB1**-DnaJ homolog subfamily B1	Chaperone	Cytosol, nucleus	Two-hybrid	[48]
**GRP75/HAPA9**- Stress-70 protein	Chaperone	Mitochondria, nucleus	Affinity Capture-Western, Two-hybrid	[48]
**SUMO4**-Small ubiquitin-related modifier 4	Protein stability	Nucleus	Affinity Capture-MS	[96]
**TBCC**- Tubulin-specific chaperone C	Protein folding chaperone	Cytosol	Two-hybrid	[48]
**TTC1**-Tetratricopeptide repeat protein 1	Protein folding	Cytosol	Two-hybrid	[48]
		**Others**		
**Aβ**-Amyloid β	Peptide	Plasma Membrane	SPR, MST,PLB	[60, 97]
**DCD**- Dermcidin	Anti-microbial activity	Secreted	Affinity Capture-MS	[49]
**ENV**-Envelope glycoprotein gp160	Virion structural	Host membrane, virion membrane	Affinity Capture-MS	[98]
**KLHL40**-Kelch-like protein 40	Muscle development	Cytosol	Two-hybrid	[99]
**Kringle 5**- K5 plasminogen domain	Angiogenic inhibitor	Secreted	BiFC, Affinity Capture-Western	[3, 4]
**PPM1G**- Protein phosphatase 1G	Serine/threonine Phosphatase	Membrane, cytosol	Two-hybrid	[48]
**SNCA**- α-synuclein	Dopamine release	Cytosol, nucleus membrane, secreted	Affinity Capture-Western	[100]
**TMEM109**-Transmembrane protein 109	UVC protection	ER, nucleus	Affinity Capture-MS	[1]
**TRAF6**- TNF receptor-associated factor 6	Activation of NF-kappa-B and JUN	Cytosol	Affinity Capture-MS	[49]
**VWA9**- von Willebrand factor A domain-containing protein 9	Unknown	Cytosol, nucleus	Affinity Capture-MS	[1]
**14-3-3**-epsilon/YWHAE	Adaptor protein	Cytosol, melanomasol	Affinity Capture-Western	[101]
**ZG16B**- Zymogen granule protein 16 homolog B	Carbohydrate binding	Secreted	Affinity Capture-MS	[33]

Supplemental References

1. Hein MY, Hubner NC, Poser I, Cox J, Nagaraj N, Toyoda Y, Gak IA, Weisswange I, Mansfeld J, Buchholz F, Hyman AA, Mann M (**2015**). A human interactome in three quantitative dimensions organized by stoichiometries and abundances. **Cell** 163(3): 712-723. doi: 10.1016/j.cell.2015.09.053

2. Tajeddine N, Galluzzi L, Kepp O, Hangen E, Morselli E, Senovilla L, Araujo N, Pinna G, Larochette N, Zamzami N, Modjtahedi N, Harel-Bellan A, Kroemer G (**2008**). Hierarchical involvement of Bak, VDAC1 and Bax in cisplatin-induced cell death. **Oncogene** 27(30): 4221-4232. doi: 10.1038/onc.2008.63

3. Li L, Yao YC, Gu XQ, Che D, Ma CQ, Dai ZY, Li C, Zhou T, Cai WB, Yang ZH, Yang X, Gao GQ (**2014**). Plasminogen kringle 5 induces endothelial cell apoptosis by triggering a voltage-dependent anion channel 1 (VDAC1) positive feedback loop. **J Biol Chem** 289(47): 32628-32638. doi: 10.1074/jbc.M114.567792

4. Gu X, Yao Y, Cheng R, Zhang Y, Dai Z, Wan G, Yang Z, Cai W, Gao G, Yang X (**2011**). Plasminogen K5 activates mitochondrial apoptosis pathway in endothelial cells by regulating Bak and Bcl-x(L) subcellular distribution. **Apoptosis** 16(8): 846-855. doi: 10.1007/s10495-011-0618-9

5. Shi Y, Chen J, Weng C, Chen R, Zheng Y, Chen Q, Tang H (**2003**). Identification of the protein-protein contact site and interaction mode of human VDAC1 with Bcl-2 family proteins. Biochem Biophys **Res Commun** 305(4): 989-996. doi. org/10.1016/S0006-291X(03)00871-4

6. Weng C, Li Y, Xu D, Shi Y, Tang H (**2005**). Specific cleavage of Mcl-1 by caspase-3 in tumor necrosis factor-related apoptosis-inducing ligand (TRAIL)-induced apoptosis in Jurkat leukemia T cells. **J Biol Chem** 280(11): 10491-500. doi: 10.1074/jbc.M412819200

7. Narita M, Shimizu S, Ito T, Chittenden T, Lutz RJ, Matsuda H, Tsujimoto Y (**1998**). Bax interacts with the permeability transition pore to induce permeability transition and cytochrome c release in isolated mitochondria. **Proc Natl Acad Sci U S A** 95(25): 14681-14686. doi: 10.1073/pnas.95.25.14681

8. Geula S, Ben-Hail D, Shoshan-Barmatz V (**2012**). Structure-based analysis of VDAC1: N-terminus location, translocation, channel gating and association with anti-apoptotic proteins. Biochem J 444(3): 475-485. doi: 10.1042/BJ20112079

9. Abu-Hamad S, Arbel N, Calo D, Arzoine L, Israelson A, Keinan N, Ben-Romano R, Friedman O, Shoshan-Barmatz V (**2009**). The VDAC1 N-terminus is essential both for apoptosis and the protective effect of anti-apoptotic proteins**. J Cell Sci** 122(Pt 11): 1906-1916. doi: 10.1242/jcs.040188

10. Arbel N, Shoshan-Barmatz V (**2010**). Voltage-dependent anion channel 1-based peptides interact with Bcl-2 to prevent antiapoptotic activity. **J Biol Chem** 285(9): 6053-6062. doi: 10.1074/jbc.M109.082990

11. Rong YP, Bultynck G, Aromolaran AS, Zhong F, Parys JB, De Smedt H, Mignery GA, Roderick HL, Bootman MD, Distelhorst CW (**2009**). The BH4 domain of Bcl-2 inhibits ER calcium release and apoptosis by binding the regulatory and coupling domain of the IP3 receptor. **Proc Natl Acad Sci U S A** 106(34): 14397-14402. doi: 10.1073/pnas.0907555106

12. Shimizu S, Narita M, Tsujimoto Y (**1999**). Bcl-2 family proteins regulate the release of apoptogenic cytochrome c by the mitochondrial channel VDAC. **Nature** 399(6735): 483-487. doi: 10.1038/20959

13. Shimizu S, Konishi A, Kodama T, Tsujimoto Y (**2000**). BH4 domain of antiapoptotic Bcl-2 family members closes voltage-dependent anion channel and inhibits apoptotic mitochondrial changes and cell death. **Proc Natl Acad Sci U S A** 97(7): 3100-3105. doi: 10.1073/pnas.97.7.3100

14.Ramanathan A, Schreiber SL (**2009**). Direct control of mitochondrial function by mTOR. **Proc Natl Acad Sci U S A** 106(52): 22229-22232. doi: 10.1073/pnas.0912074106

15.Huang H, Hu X, Eno CO, Zhao G, Li C, White C (**2013**). An interaction between Bcl-xL and the voltage-dependent anion channel (VDAC) promotes mitochondrial Ca2+ uptake. **J Biol Chem** 288(27): 19870-19881. doi: 10.1074/jbc.M112.448290

16. Sugiyama T, Shimizu S, Matsuoka Y, Yoneda Y, Tsujimoto Y (**2002**). Activation of mitochondrial voltage-dependent anion channel by apro-apoptotic BH3-only protein Bim. **Oncogene** 21(32): 4944-4956. doi: 10.1038/sj.onc.1205621

17. Gao Z, Shang Q, Liu Z, Deng C, Guo C (**2015**). Mitochondrial ATF2 translocation contributes to apoptosis induction and BRAF inhibitor resistance in melanoma through the interaction of Bim with VDAC1. **Oncotarget** 6(34): 36338-36353. doi: 10.18632/oncotarget.5537

18. Brahimi-Horn MC, Lacas-Gervais S, Adaixo R, Ilc K, Rouleau M, Notte A, Dieu M, Michiels C, Voeltzel T, Maguer-Satta V, Pelletier J, Ilie M, Hofman P, Manoury B, Schmidt A, Hiller S, Pouyssegur J, Mazure NM (**2015**). Local mitochondrial-endolysosomal microfusion cleaves voltage-dependent anion channel 1 to promote survival in hypoxia. **Mol Cell Biol** 35(9): 1491-1505. doi: 10.1128/MCB.01402-14

19. Zhang X, Weng C, Li Y, Wang X, Jiang C, Li X, Xu Y, Chen Q, Pan L, Tang H (**2012**). Human Bop is a novel BH3-only member of the Bcl-2 protein family. **Protein Cell** 3(10): 790-801 .doi: 10.1007/s13238-012-2069-7

19. Pazarentzos E, Mahul-Mellier AL, Datler C, Chaisaklert W, Hwang MS, Kroon J, Qize D, Osborne F, Al-Rubaish A, Al-Ali A, Mazarakis ND, Aboagye EO, Grimm S (**2014**). IkappaBetaalpha inhibits apoptosis at the outer mitochondrial membrane independently of NF-kappaB retention. **EMBO J** 33(23): 2814-2828. doi: 10.15252/embj.201488183

20. Rostovtseva TK, Antonsson B, Suzuki M, Youle RJ, Colombini M, Bezrukov SM (**2004**). Bid, but not Bax, regulates VDAC channels. **J Biol Chem** .13583-13575 :(14)279. doi: 10.1074/jbc.M310593200

21. Chen Y, Yang LN, Cheng L, Tu S, Guo SJ, Le HY, Xiong Q, Mo R, Li CY, Jeong JS, Jiang L, Blackshaw S, Bi LJ, Zhu H, Tao SC, Ge F (**2013**). Bcl2-associated athanogene 3 interactome analysis reveals a new role in modulating proteasome activity. **Mol Cell Proteomics** 12(10): 2804-2819. doi: 10.1074/mcp.M112.025882

22. Arzoine L, Zilberberg N, Ben-Romano R, Shoshan-Barmatz V (**2009**). Voltage-dependent anion channel 1-based peptides interact with hexokinase to prevent its anti-apoptotic activity. **J Biol Chem** 284(6): 3946-3955. doi: 10.1074/jbc.M803614200

23. 8Arzoine L, Zilberberg N, Ben-Romano R, Shoshan-Barmatz V (**2009**). Voltage-dependent anion channel 1-based peptides interact with hexokinase to prevent its anti-apoptotic activity. **J Biol Chem** 284(6): 3946-3955. doi: 10.1074/jbc.M803614200

24. Lau E, Kluger H, Varsano T, Lee K, Scheffler I, Rimm DL, Ideker T, Ronai ZA (**2012**). PKCepsilon promotes oncogenic functions of ATF2 in the nucleus while blocking its apoptotic function at mitochondria. **Cell** 148(3): 543-555. doi: 10.1016/j.cell.2012.01.016

25. Blachly-Dyson E, Zambronicz EB, Yu WH, Adams V, McCabe ER, Adelman J, Colombini M, Forte M (**1993**). Cloning and functional expression in yeast of two human isoforms of the outer mitochondrial membrane channel, the voltage-dependent anion channel. **J Biol Chem** 268(3): 1835-1841. PMID: 8420959

26. Shoshan-Barmatz V, Israelson A (**2005**). The voltage-dependent anion channel in endoplasmic/sarcoplasmic reticulum: characterization, modulation and possible function. **J Membr Biol** 204(2): 57-66. doi: 10.1007/s00232-005-0749-4

27. Marzo I, Brenner C, Zamzami N, Susin SA, Beutner G, Brdiczka D, Remy R, Xie ZH, Reed JC, Kroemer G (**1998**). The permeability transition pore complex: a target for apoptosis regulation by caspases and bcl-2-related proteins. **J Exp Med** 187(8): 1261-1271. doi: 10.1084/jem.187.8.1261

28. Allouche M, Pertuiset C, Robert JL, Martel C, Veneziano R, Henry C, dein OS, Saint N, Brenner C, Chopineau J (**2012**). ANT-VDAC1 interaction is direct and depends on ANT isoform conformation in vitro. **Biochem Biophys Res Commun** 429(1-2): 12-17. doi: 10.1016/j.bbrc.2012.10.108

29. Lee K, Kerner J, Hoppel CL (**2011**). Mitochondrial carnitine palmitoyltransferase 1a (CPT1a) is part of an outer membrane fatty acid transfer complex. **J Biol Chem** 286(29): 25655-62. doi: 10.1074/jbc.M111.228692

30. Vyssokikh MY, Zorova L, Zorov D, Heimlich G, Jurgensmeier JJ, Brdiczka D (**2002**). Bax releases cytochrome c preferentially from a complex between porin and adenine nucleotide translocator. Hexokinase activity suppresses this effect. **Mol Biol Rep** 29(1-2): 93-96. PMID: 12243190

31.Tarze A, Deniaud A, Le Bras M, Maillier E, Molle D, Larochette N, Zamzami N, Jan G, Kroemer G, Brenner C (**2007**). GAPDH, a novel regulator of the pro-apoptotic mitochondrial membrane permeabilization. **Oncogene** 26(18): 2606-2620. doi: 10.1038/sj.onc.1210074

32. Adams V, Griffin L, Towbin J, Gelb B, Worley K, McCabe ER (**1991**). Porin interaction with hexokinase and glycerol kinase: metabolic microcompartmentation at the outer mitochondrial membrane. **Biochem Med Metab Biol** 45(3): 271-291. doi: 10.1016/0885-4505(91)90032-G

33. Huttlin EL, Ting L, Bruckner RJ, Gebreab F, Gygi MP, Szpyt J, Tam S, Zarraga G, Colby G, Baltier K, Dong R, Guarani V, Vaites LP, Ordureau A, Rad R, Erickson BK, Wuhr M, Chick J, Zhai B, Kolippakkam D, Mintseris J, Obar RA, Harris T, Artavanis-Tsakonas S, Sowa ME, De Camilli P, Paulo JA, Harper JW, Gygi SP (**2015**). The BioPlex Network: A Systematic Exploration of the Human Interactome. **Cell** 162(2): 425-440. doi: 10.1016/j.cell.2015.06.043

34. Schlattner U, Dolder M, Wallimann T, Tokarska-Schlattner M (**2001**). Mitochondrial creatine kinase and mitochondrial outer membrane porin show a direct interaction that is modulated by calcium. **J Biol Chem** 276(51): 48027-48030. doi: 10.1074/jbc.M106524200

35. Brdiczka D, Kaldis P, Wallimann T (**1994**). In vitro complex formation between the octamer of mitochondrial creatine kinase and porin. **J Biol Chem** 269(44): 27640-27644. PMID: 7525559

36. Bose M, Whittal RM, Miller WL, Bose HS (**2008**). Steroidogenic activity of StAR requires contact with mitochondrial VDAC1 and phosphate carrier protein. **J Biol Chem** 283(14): 8837-8845. doi: 10.1074/jbc.M709221200

37. Veenman L, Papadopoulos V, Gavish M (**2007**). Channel-like functions of the 18-kDa translocator protein (TSPO): regulation of apoptosis and steroidogenesis as part of the host-defense response. **Curr Pharm Des** 13(23): 2385-2405. doi: 10.2174/138161207781368710

38. Gatliff J, East D, Crosby J, Abeti R, Harvey R, Craigen W, Parker P, Campanella M (**2014**). TSPO interacts with VDAC1 and triggers a ROS-mediated inhibition of mitochondrial quality control. **Autophagy** 10(12): 2279-2296. doi: 10.4161/15548627.2014.991665

39. Crompton M, Virji S, Ward JM (**1998**). Cyclophilin-D binds strongly to complexes of the voltage-dependent anion channel and the adenine nucleotide translocase to form the permeability transition pore. **Eur J Biochem** 258(2): 729-735. doi: 10.1046/j.1432-1327.1998.2580729.x

40. Jackson JG, O'Donnell JC, Krizman E, Robinson MB (**2015**). Displacing hexokinase from mitochondrial voltage-dependent anion channel impairs GLT-1-mediated glutamate uptake but does not disrupt interactions between GLT-1 and mitochondrial proteins. **J Neurosci Res** 93(7): 999-1008. doi: 10.1002/jnr.23533

41. Castello A, Fischer B, Eichelbaum K, Horos R, Beckmann BM, Strein C, Davey NE, Humphreys DT, Preiss T, Steinmetz LM, Krijgsveld J, Hentze MW (**2012**). Insights into RNA biology from an atlas of mammalian mRNA-binding proteins. **Cell** 149(6): 1393-1406. doi: 10.1016/j.cell.2012.04.031

42. Richter R, Rorbach J, Pajak A, Smith PM, Wessels HJ, Huynen MA, Smeitink JA ,Lightowlers RN, Chrzanowska-Lightowlers ZM (**2010**). A functional peptidyl-tRNA hydrolase, ICT1, has been recruited into the human mitochondrial ribosome. **EMBO J** 29(6): 1116-1125. doi: 10.1038/emboj.2010.14

43. Guan K, Zheng Z, Song T, He X, Xu C, Zhang Y, Ma S, Wang Y, Xu Q, Cao Y, Li J, Yang X, Ge X, Wei C, Zhong H (**2013**). MAVS regulates apoptotic cell death by decreasing K48-linked ubiquitination of voltage-dependent anion channel 1. **Mol Cell Biol** 33(16): 3137-3149. doi: 10.1128/MCB.00030-13

44. Fu F, Wu D, Qian C (**2016**). The MicroRNA-2 24Inhibitor Prevents Neuronal Apoptosis via Targeting Spastic Paraplegia 7 After Cerebral Ischemia. **J Mol Neurosci** 59(3): 421-429. doi: 10.1007/s12031-016-0769-9

45. Schleiff E, Shore GC, Goping IS (**1997**). Human mitochondrial import receptor, Tom20p. Use of glutathione to reveal specific interactions between Tom20-glutathione S-transferase and mitochondrial precursor proteins. **FEBS Lett** 404(2-3): 314-318. doi: 10.1016/s0014-5793(97)00145-2

46. Alvira CM, Umesh A, Husted C, Ying L, Hou Y, Lyu SC, Nowak J, Cornfield DN (**2012**). Voltage-dependent anion channel-2 interaction with nitric oxide synthase enhances pulmonary artery endothelial cell nitric oxide production. **Am J Respir Cell Mol Biol** 47(5): 669-678. doi: 10.1165/rcmb.2011-0436OC

47. Israelson A, Arbel N, Da Cruz S, Ilieva H, Yamanaka K, Shoshan-Barmatz V, Cleveland DW (**2010**). Misfolded mutant SOD1 directly inhibits VDAC1 conductance in a mouse model of inherited ALS. **Neuron** 67(4): 575-587. doi: 10.1016/j.neuron.2010.07.019

48. Szabadkai G, Bianchi K, Varnai P, De Stefani D, Wieckowski MR, Cavagna D, Nagy AI, Balla T, Rizzuto R (**2006**). Chaperone-mediated coupling of endoplasmic reticulum and mitochondrial Ca2+ channels. **J Cell Biol** 175(6): 901-911. doi: 10.1083/jcb.200608073

49.Ewing RM, Chu P, Elisma F, Li H, Taylor P, Climie S, McBroom-Cerajewski L, Robinson MD, O'Connor L, Li M, Taylor R, Dharsee M, Ho Y, Heilbut A, Moore L, Zhang S, Ornatsky O, Bukhman YV, Ethier M, Sheng Y, Vasilescu J, Abu-Farha M, Lambert JP, Duewel HS, Stewart, II, Kuehl B, Hogue K, Colwill K, Gladwish K, Muskat B, et al. (**2007**). Large-scale mapping of human protein-protein interactions by mass spectrometry. **Mol Syst Biol** 3:89. doi: 10.1038/msb4100134

50. Hur JY, Teranishi Y, Kihara T, Yamamoto NG, Inoue M, Hosia W, Hashimoto M, Winblad B, Frykman S, Tjernberg LO (**2012**). Identification of novel gamma-secretase-associated proteins in detergent-resistant membranes from brain. **J Biol Chem** 287(15): 11991-12005. doi: 10.1074/jbc.M111.246074

51. Neganova I, Vilella F, Atkinson SP, Lloret M, Passos JF, von Zglinicki T, O'Connor JE, Burks D, Jones R, Armstrong L, Lako M (**2011**). An important role for CDK2 in G1 to S checkpoint activation and DNA damage response in human embryonic stem cells. **Stem Cells** 29(4): 651-9. doi: 10.1002/stem.620

52. Vinayagam A, Stelzl U, Foulle R, Plassmann S, Zenkner M, Timm J, Assmus HE, Andrade-Navarro MA, Wanker EE (**2011**). A directed protein interaction network for investigating intracellular signal transduction. **Sci Signal** 4(189): rs8. doi: 10.1126/scisignal.2001699

53. Galli S, Jahn O, Hitt R, Hesse D, Opitz L, Plessmann U, Urlaub H, Poderoso JJ, Jares-Erijman EA, Jovin TM (**2009**). A new paradigm for MAPK: structural interactions of hERK1 with mitochondria in HeLa cells. **PLoS One** 4(10): e7541. doi: 10.1371/journal.pone.0007541

54. Zhang X, Ling Y, Guo Y, Bai Y, Shi X, Gong F, Tan P, Zhang Y, Wei C, He X, Ramirez A, Liu X, Cao C, Zhong H, Xu Q, Ma RZ (**2016**). Mps1 kinase regulates tumor cell viability via its novel role in mitochondria. **Cell Death Dis** 7(7): e2292. doi: 10.1038/cddis.2016.193

55. Chen Y, Craigen WJ, Riley DJ (**2009**). Nek1 regulates cell death and mitochondrial membrane permeability through phosphorylation of VDAC1. **Cell Cycle** 8(2): 257-67. doi: 10.4161/cc.8.2.7551

56. Chen Y, Gaczynska M, Osmulski P, Polci R, Riley DJ (**2010**). Phosphorylation by Nek1 regulates opening and closing of voltage dependent anion channel 1. **Biochem Biophys Res Commun** 394(3): 798-803. doi: 10.1016/j.bbrc.2010.03.077

57. Baines CP, Song CX, Zheng YT, Wang GW, Zhang J, Wang OL, Guo Y, Bolli R, Cardwell EM, Ping P (**2003**). Protein kinase Cepsilon interacts with and inhibits the permeability transition pore in cardiac mitochondria. **Circ Res** 92(8): 873-880. doi: 10.1161/01.RES.0000069215.36389.8D

58. Le Mellay V, Troppmair J, Benz R, Rapp UR (**2002**). Negative regulation of mitochondrial VDAC channels by C-Raf kinase. **BMC Cell Biol** 3:14. doi: 10.1186/1471-2121-3-

59. Kittanakom S, Barrios-Rodiles M, Petschnigg J, Arnoldo A, Wong V, Kotlyar M, Heisler LE, Jurisica I, Wrana JL, Nislow C, Stagljar I (**2014**). CHIP-MYTH: a novel interactive proteomics method for the assessment of agonist-dependent interactions of the human beta(2)-adrenergic receptor. **Biochem Biophys Res Commun** 445(4): 746-756. doi: 10.1016/j.bbrc.2014.02.033

60. Fernandez-Echevarria C, Diaz M, Ferrer I, Canerina-Amaro A, Marin R (**2014**). Abeta promotes VDAC1 channel dephosphorylation in neuronal lipid rafts. Relevance to the mechanisms of neurotoxicity in Alzheimer's disease. **Neuroscience** 278: 354-366. doi: 10.1016/j.neuroscience.2014.07.079

61. Bernhard OK, Cunningham AL, Sheil MM (**2004**). Analysis of proteins copurifying with the CD4/lck complex using one-dimensional polyacrylamide gel electrophoresis and mass spectrometry: comparison with affinity-tag based protein detection and evaluation of different solubilization methods. **J Am Soc Mass Spectrom** 15(4): 558-567. doi: 10.1016/j.jasms.200.3.12.006

62. Gincel D, Zaid H, Shoshan-Barmatz V (**2001**). Calcium binding and translocation by the voltage-dependent anion channel: a possible regulatory mechanism in mitochondrial function. **Biochem J** 358(Pt 1): 147-155. doi: 10.1042/0264-6021:3580147

63. Humphries JD, Byron A, Bass MD, Craig SE, Pinney JW, Knight D, Humphries MJ (**2009**). Proteomic analysis of integrin-associated complexes identifies RCC2 as a dual regulator of Rac1 and Arf6. **Sci Signal** 2(87): ra51. doi: 10.1126/scisignal.20.00396

64. Havugimana PC, Hart GT, Nepusz T, Yang H, Turinsky AL, Li Z, Wang PI, Boutz DR, Fong V, Phanse S, Babu M, Craig SA, Hu P, Wan C, Vlasblom J, Dar VU, Bezginov A, Clark GW, Wu GC, Wodak SJ, Tillier ER, Paccanaro A, Marcotte EM, Emili A (**2012**). A census of human soluble protein complexes. **Cell** 150(5): 1068-1081. doi: 10.1016/j.cell.2012.08.011

65.Wan C, Borgeson B, Phanse S, Tu F, Drew K, Clark G, Xiong X, Kagan O, Kwan J, Bezginov A, Chessman K, Pal S, Cromar G, Papoulas O, Ni Z, Boutz DR, Stoilova S, Havugimana PC, Guo X, Malty RH, Sarov M, Greenblatt J, Babu M, Derry WB, Tillier ER, Wallingford JB, Parkinson J, Marcotte EM, Emili A (**2015**). Panorama of ancient metazoan macromolecular complexes. **Nature** 525(7569): 339-344. doi: 10.1038/nature14877

66. Roman I, Figys J, Steurs G, Zizi M (**2006**). Direct measurement of VDAC-actin interaction by surface plasmon resonance. **Biochim Biophys Acta** 1758(4): 479-486. doi: 10.1016/j.bbamem.2006.03.019

67. Xu X, Forbes JG, Colombini M (**2001**). Actin modulates the gating of Neurospora crassa VDAC. **J Membr Biol** 180(1): 73-81. doi: 10.1007/s002320010060

68. Ramirez CM, Gonzalez M, Diaz M, Alonso R, Ferrer I, Santpere G, Puig B, Meyer G, Marin R (**2009**). VDAC and ERalpha interaction in caveolae from human cortex is altered in Alzheimer's disease. **Mol Cell Neurosci** 42(3): 172-183. doi: 10.1016/j.mcn.2009.07.001

69. Schwarzer C, Barnikol-Watanabe S, Thinnes FP, Hilschmann N (**2002**). Voltage-dependent anion-selective channel (VDAC) interacts with the dynein light chain Tctex1 and the heat-shock protein PBP74. **Int J Biochem Cell Biol** 34(9): 1059-1070. doi: 10.1016/s1357-2725(02)00026-2

70. Han Y, Yu G, Sarioglu H, Caballero-Martinez A, Schlott F, Ueffing M, Haase H, Peschel C, Krackhardt AM (**2013**). Proteomic investigation of the interactome of FMNL1 in hematopoietic cells unveils a role in calcium-dependent membrane plasticity .**J Proteomics** 78:72-82. doi: 10.1016/j.jprot.2012.11.015

71. Kusano H, Shimizu S, Koya RC, Fujita H, Kamada S, Kuzumaki N, Tsujimoto Y (**2000**). Human gelsolin prevents apoptosis by inhibiting apoptotic mitochondrial changes via closing VDAC. **Oncogene** 19(42): 4807-4814. doi: 10.1038/sj.onc.1203868

72. Hutchins JR, Toyoda Y, Hegemann B, Poser I, Heriche JK, Sykora MM, Augsburg M, Hudecz O, Buschhorn BA, Bulkescher J, Conrad C, Comartin D, Schleiffer A, Sarov M, Pozniakovsky A, Slabicki MM, Schloissnig S, Steinmacher I, Leuschner M, Ssykor A, Lawo S, Pelletier L, Stark H, Nasmyth K, Ellenberg J, Durbin R, Buchholz F, Mechtler K, Hyman AA, Peters JM (**2010**). Systematic analysis of human protein complexes identifies chromosome segregation proteins. **Science** 328(5978): 593-599. doi: 10.1126/science.1181348

73. Lindén M, Karlsson G (**1996**). Identification of Porin as a Binding Site for MAP2. **Biochemical and Biophysical Research Communications** 218(3): 833-836. doi: 10.1006/bbrc.1996.0148

74. Miura N, Takemori N, Kikugawa T, Tanji N, Higashiyama S, Yokoyama M (**2012**). Adseverin: a novel cisplatin-resistant marker in the human bladder cancer cell line HT1376 identified by quantitative proteomic analysis. **Mol Oncol** 6(3): 311-322 .doi: 10.1016/j.molonc.2011.12.002

75. Carre M, Andre N, Carles G, Borghi H, Brichese L, Briand C, Braguer D (**2002**). Tubulin is an inherent component of mitochondrial membranes that interacts with the voltage-dependent anion channel. **J Biol Chem** 277(37): 33664-33669. doi: 10.1074/jbc.M203834200

76. Maldonado EN, Patnaik J, Mullins MR, Lemasters JJ (**2010**). Free tubulin modulates mitochondrial membrane potential in cancer cells. **Cancer Res** 70(24): 10192-10201. doi: 10.1158/0008-5472.CAN-10-2429

77. Koppenol WH, Bounds PL, Dang CV (**2011**). Otto Warburg's contributions to current concepts of cancer metabolism. **Nat Rev Cancer** 11(5): 325-337. doi: 10.1038/nrc3038

78. Andresen CA, Smedegaard S, Sylvestersen KB, Svensson C, Iglesias-Gato D, Cazzamali G, Nielsen TK, Nielsen ML, Flores-Morales A (**2014**). Protein interaction screening for the ankyrin repeats and suppressor of cytokine signaling (SOCS) box (ASB) family identify Asb11 as a novel endoplasmic reticulum resident ubiquitin ligase. **J Biol Chem** 289(4): 2043-2054. doi: 10.1074/jbc.M113.534602

79. Christianson JC, Olzmann JA, Shaler TA, Sowa ME, Bennett EJ, Richter CM, Tyler RE, Greenblatt EJ, Harper JW, Kopito RR (**2012**). Defining human ERAD networks through an integrative mapping strategy. **Nat Cell Biol** 14(1): 93-105. doi: 10.1038/ncb2383

80. Okatsu K, Iemura S, Koyano F, Go E, Kimura M, Natsume T, Tanaka K, Matsuda N .(2012) Mitochondrial hexokinase HKI is a novel substrate of the Parkin ubiquitin ligase. **Biochem Biophys Res Commun** 428(1): 197-202. doi: 10.1016/j.bbrc.2012.10.041

81. Sun Y, Vashisht AA, Tchieu J, Wohlschlegel JA, Dreier L (**2012**). Voltage-dependent anion channels (VDACs) recruit Parkin to defective mitochondria to promote mitochondrial autophagy. **J Biol Chem** 287(48): 40652-40660. doi: 10.1074/jbc.M112.419721

82. Sarraf SA, Raman M, Guarani-Pereira V, Sowa ME, Huttlin EL, Gygi SP, Harper JW (**2013**) Landscape of the PARKIN-dependent ubiquitylome in response to mitochondrial depolarization. **Nature** 496(7445): 372-376. doi: 10.1038/nature12043

83. Ham SJ, Lee SY, Song S, Chung JR, Choi S, Chung J (**2016**). Interaction between RING1 (R1) and the Ubiquitin-like (UBL) Domains Is Critical for the Regulation of Parkin Activity. **J Biol Chem** 291(4): 1803-1816. doi: 10.1074/jbc.M115.687319

84. Danielsen JM, Sylvestersen KB, Bekker-Jensen S, Szklarczyk D, Poulsen JW, Horn H, Jensen LJ, Mailand N, Nielsen ML (**2011**). Mass spectrometric analysis of lysine ubiquitylation reveals promiscuity at site level. **Mol Cell Proteomics** 10(3): M110 003590. doi: 10.1074/mcp.M110.003590

85. Matsumoto M, Hatakeyama S, Oyamada K, Oda Y, Nishimura T, Nakayama KI (**2005**). Large-scale analysis of the human ubiquitin-related proteome. **Proteomics** 5(16): 4145-4151. doi: 10.1002/pmic.200401280

86. Wagner SA, Beli P, Weinert BT, Nielsen ML, Cox J, Mann M, Choudhary C (**2011**). A proteome-wide, quantitative survey of in vivo ubiquitylation sites reveals widespread regulatory roles. **Mol Cell Proteomics** 10(10): M111 013284. doi: 10.1074/mcp.M111.013284

87. Kim W, Bennett EJ, Huttlin EL, Guo A, Li J, Possemato A, Sowa ME, Rad R, Rush J, Comb MJ, Harper JW, Gygi SP (**2011**). Systematic and quantitative assessment of the ubiquitin-modified proteome. **Mol Cell** 44(2): 325-340. doi: 10.1016/j.molcel.2011.08.025

88. Greco TM, Yu F, Guise AJ, Cristea IM (**2011**). Nuclear import of histone deacetylase 5 by requisite nuclear localization signal phosphorylation. **Mol Cell Proteomics** 10(2): M110 004317. doi: 10.1074/mcp.M110.004317

89. Woods NT, Mesquita RD, Sweet M, Carvalho MA, Li X, Liu Y, Nguyen H, Thomas CE, Iversen ES, Jr., Marsillac S, Karchin R, Koomen J, Monteiro AN (**2012**). Charting the landscape of tandem BRCT domain-mediated protein interactions. **Sci Signal** 5(242): rs6. doi: 10.1126/scisignal.2002255

90. Lambert JP, Tucholska M, Go C, Knight JD, Gingras AC (**2015**). Proximity biotinylation and affinity purification are complementary approaches for the interactome mapping of chromatin-associated protein complexes. **J Proteomics** 118:81-94. doi: 10.1016/j.jprot.2014.09.011

91. Tsai YC, Greco TM, Boonmee A, Miteva Y, Cristea IM (**2012**). Functional proteomics establishes the interaction of SIRT7 with chromatin remodeling complexes and expands its role in regulation of RNA polymerase I transcription. **Mol Cell Proteomics** 11(5): 60-76. doi: 10.1074/mcp.A111.015156

92. Kim MS, Machida Y, Vashisht AA, Wohlschlegel JA, Pang YP, Machida YJ (**2013**). Regulation of error-prone translesion synthesis by Spartan/C1orf124. **Nucleic Acids Res** 41(3): 1661-1668. doi: 10.1093/nar/gks1267

93. Elzi DJ, Song M, Hakala K, Weintraub ST, Shiio Y (**2014**). Proteomic Analysis of the EWS-Fli-1 Interactome Reveals the Role of the Lysosome in EWS-Fli-1 Turnover. **J Proteome Res** 13(8): 3783-3791. doi: 10.1021/pr500387m

94. Emdal KB, Pedersen AK, Bekker-Jensen DB, Tsafou KP, Horn H, Lindner S, Schulte JH, Eggert A, Jensen LJ, Francavilla C, Olsen JV (**2015**). Temporal proteomics of NGF-TrkA signaling identifies an inhibitory role for the E3 ligase Cbl-b in neuroblastoma cell differentiation. **Sci Signal** 8(374): ra40. doi: 10.1126/scisignal.2005769

95. Mitra A, Basak T, Datta K, Naskar S, Sengupta S, Sarkar S (**2013**). Role of alpha-crystallin B as a regulatory switch in modulating cardiomyocyte apoptosis by mitochondria or endoplasmic reticulum during cardiac hypertrophy and myocardial infarction. **Cell death & disease** 4:e582. doi: 10.1038/cddis.2013.114

96. Guo D, Han J, Adam BL, Colburn NH, Wang MH, Dong Z, Eizirik DL, She JX, Wang CY (**2005**). Proteomic analysis of SUMO4 substrates in HEK293 cells under serum starvation-induced stress. **Biochem Biophys Res Commun** 337(4): 1308-1318. doi: 10.1016/j.bbrc.2005.09.191

97. Smilansky A, Dangoor L, Nakdimon I, Ben-Hail D, Mizrachi D, Shoshan-Barmatz V (**2015**). The Voltage-dependent Anion Channel 1 Mediates Amyloid beta Toxicity and Represents a Potential Target for Alzheimer Disease Therapy. **J Biol Chem** 290(52): 30670-30683. doi: 10.1074/jbc.M115.691493

98. Jager S, Cimermancic P, Gulbahce N, Johnson JR, McGovern KE, Clarke SC, Shales M, Mercenne G, Pache L, Li K, Hernandez H, Jang GM, Roth SL, Akiva E, Marlett J, Stephens M, D'Orso I, Fernandes J, Fahey M, Mahon C, O'Donoghue AJ, Todorovic A, Morris JH, Maltby DA, Alber T, Cagney G, Bushman FD, Young JA ,Chanda SK, Sundquist WI, et al. (**2012**). Global landscape of HIV-human protein complexes. **Nature** 481(7381): 365-370. doi: 10.1038/nature10719

99. Garg A, O'Rourke J, Long C, Doering J, Ravenscroft G, Bezprozvannaya S, Nelson BR, Beetz N, Li L, Chen S, Laing NG, Grange RW, Bassel-Duby R, Olson EN (**2014**). KLHL40 deficiency destabilizes thin filament proteins and promotes nemaline myopathy. **J Clin Invest** 124(8): 3529-3539. doi: 10.1172/JCI74994

100. McFarland MA, Ellis CE, Markey SP, Nussbaum RL (**2008**) .Proteomics analysis identifies phosphorylation-dependent alpha-synuclein protein interactions. **Mol Cell Proteomics** 7(11): 2123-2137. doi: 10.1074/mcp.M800116-MCP200

101. Aghazadeh Y, Martinez-Arguelles DB, Fan J, Culty M, Papadopoulos V (**2014**). Induction of androgen formation in the male by a TAT-VDAC1 fusion peptide blocking 14-3-3ε protein adaptor and mitochondrial VDAC1 interactions. **Mol Ther** 22(10): 1779-91. doi: 10.1038/mt.2014.116
